# Synthesis and Characterization of New Series of 1,3-5-Triazine Hydrazone Derivatives with Promising Antiproliferative Activity

**DOI:** 10.3390/molecules25112708

**Published:** 2020-06-11

**Authors:** Hessa H. Al Rasheed, Azizah M. Malebari, Kholood A. Dahlous, Ayman El-Faham

**Affiliations:** 1 Department of Chemistry, College of Science, King Saud University, P.O. Box 2455, Riyadh 11451, Saudi Arabia; kdahloos@KSU.EDU.SA; 2 Department of Pharmaceutical Chemistry, College of Pharmacy, King Abdulaziz University, P.O. Box 80260, Jeddah 21589, Saudi Arabia; amelibary@kau.edu.sa; 3 Chemistry Department, Faculty of Science, Alexandria University, P.O. Box 426, Ibrahimia, Alexandria 12321, Egypt

**Keywords:** s-Triazine, hydrazone derivatives, antiproliferative activity, MCF-7, HCT-116

## Abstract

A new series of *s*-triazine hydrazone derivatives was prepared based on the reaction of 6-hydrazino-2,4-disubstituted-*s*-triazine with *p*-substituted benzaldehyde derivatives using a straightforward synthetic pathway. The antiproliferative activity of all synthesized compounds was evaluated against two human cancer cell lines; breast cancer MCF-7 and colon carcinoma HCT-116 using MTT assay. Among all, 11 compounds have shown strong to moderate antiproliferative activity with IC_50_ values in the range 1.01–18.20 µM in MCF-7 and 0.97–19.51 µM in HCT-116. The best results were obtained with 4,4’-(6-(2-(pyridin-2-ylmethylene)hydrazinyl)-1,3,5-triazine-2,4-diyl) dimorpholine **11** (IC_50_ = 1.0 µM and 0.98 µM in MCF-7 and HCT-116 cell lines, respectively). The substituents on the *s-*triazine core as well as the substituent at the benzylidene moiety have a great effect on the antiproliferative activity. Whereas compounds containing dimorpholino-*s*-triazine derivatives **8a–e** showed more potent antiproliferative in MCF-7 compared to their analogs **7a–f** (compounds containing two-piperidine rings), compounds containing one piperidine and one morpholine ring **9a–f** showed better IC_50_ values in the range 10.4–22.2 µM. On the other hand, compounds containing two-piperidine rings **7a–f** showed more potent antiproliferative in HCT-116 (IC_50_ values in the range 8.8–19.5 µM) than their analogs **8a–e** and **9a–f**.

## 1. Introduction

*s*-Triazine (1,3,5-triazine) is a heterocyclic compound, which is quite stable and its derivatives have wide practical applications in numerous fields. The most important reagent for obtaining these derivatives is cyanuric chloride (2,4-6-trichloro-1,3,5-triazine). This compound has provoked significant consideration due to its low cost, commercial availability, and considered as an ideal starting material in stepwise nucleophilic substitution for the synthesis of symmetric and nonsymmetric *s*-triazine derivatives at different temperatures [1,2,3].

The *s*-triazine scaffold affords the basis for the design and synthesis of various biologically active compounds with widespread applications in medicinal chemistry [4,5,6,7,8,9,10,11]. On the other hand, many researchers made notable progress on the design, synthesis, and evaluation of numerous *s*-triazine derivatives with great and promising results for the further development of new antitumor agents [12,13,14,15,16,17,18,19,20].

**Figure 1:**
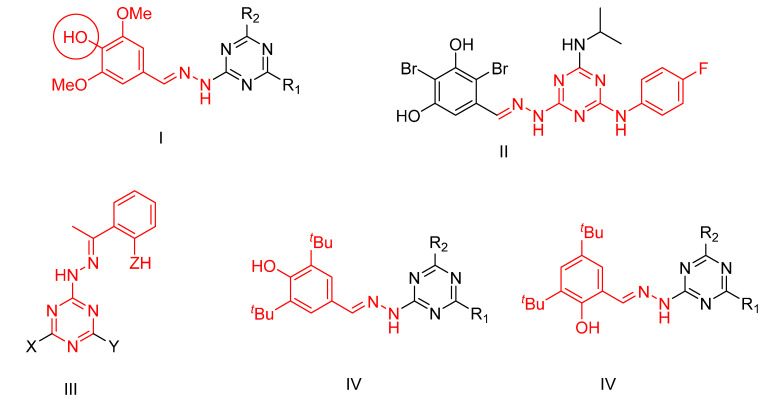
Structure of the *s*-triazine hydrazone derivative with potent anticancer activity.

Recently, a series of *s*-triazine hydrazino derivatives **I** (Figure 1) was reported as selective inhibitors of the mammalian target of rapamycin (mTOR) [21]. The results showed that the phenolic hydroxyl group has a critical effect, while its analogs methoxy derivative showed a dramatic loss in the activity [21]. Later, another series of *s*-triazine hydrazone derivatives was reported with their particular target inhibitors to both epidermal growth factor (EGFR) and mutant epidermal growth factor tyrosine kinase receptors (EGFR TKs) [22]. Of these derivatives, the fluoro derivative **II** (Figure 1) showed the most potent activity against epidermal growth factor receptor (EGFR). Moreover, it exhibited considerable antiproliferative activity against A549, A431, and NCIH1975 cell lines.

In a recent study [23], we reported the synthesis and evaluation of the antiproliferative activities of several *s*-triazine hydrazone derivatives **III** (Figure 1). The results showed that the substituents on the *s*-triazine ring especially, methoxy group and piperidine ring conferred greater selectivity for human liver cancer cell lines (HepG2), while the presence of the morpholine and piperidine ring exhibited greater selectivity for adenocarcinomic human alveolar basal epithelial cells (A549) with a reasonable inhibitory effect on HepG2 cells [23].

Later, we reported another series of *s*-triazine hydrazone derivatives that encompasses *s*-triazine and (2 or 4)-hydroxylbenzylidene derivatives **IV** and **V** (Figure 1) with their anticancer activity [24]. The results revealed that the position of the hydroxyl group on the benzylidine ring as well as the substituent on the *s*-triazine moiety have a great effect on the anticancer activity against breast cancer cells (MCF-7) and colon cancer (HCT-116).

Based on the reported results by our group and others, *s*-triazine, morpholine, and piperidine specifically serve as templates of a number of clinically used drugs [25], we report here the synthesis of a small library of compounds bearing the *s*-triazine, morpholine and piperidine core with benzylidene derivatives through the hydrazone linkage. The antiproliferative activity of this new series against breast cancer MCF-7 and colon carcinoma HCT-116 cell lines were evaluated as well.

## 2. Results and Discussion

### 2.1. Chemistry

**Scheme 1:**
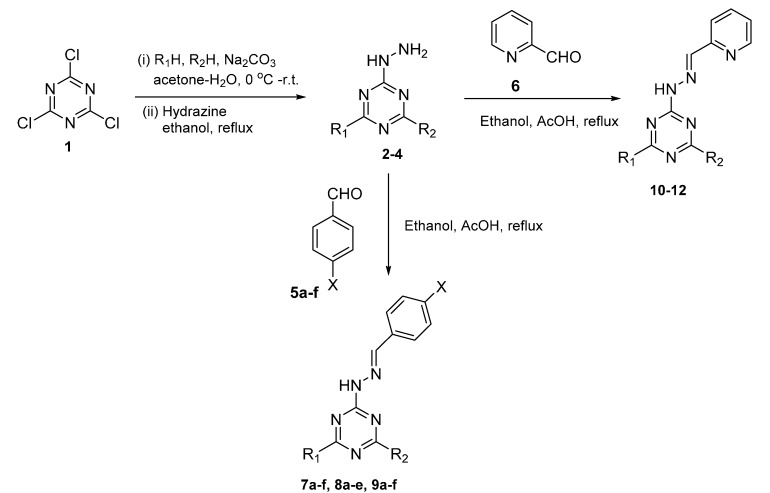
Synthetic route for preparation of the target products.

The target products **7a–f**, **8a–e**, **9a–f**, and **10–12** were synthesized in three steps as follows. As a first step, cyanuric chloride was reacted with different amines according to the reported method [26] to afford 2-chloro-4,6-disubstituted-*s*-triazine derivatives in good yields and purities. The spectral data were in good agreement with the reported ones in literature [26]. As a second step, 2-chloro-4,6-disubstituted-*s*-triazine derivatives reacted with hydrazine hydrate in ethanol following the reported method [26,27] to afford the hydrazino derivatives **2–4** as a white solid in good yields and purities which have been used directly into the next step. Finally, 6-hydrazino-2,4-disubstituted-*s*-triazine derivatives **2–4** were reacted with *p-*substituted benzaldehyde derivatives **5a–f** or 2-pyridinecarboxaldehyde **6** in the presence of the catalytic amount of acetic acid (AcOH) and ethanol as solvent to give the target products **7a–f**, **8a–e**, **9a–f**, and **10–12** (Scheme 1, Table 1). The structure of the target products was confirmed by elemental analysis, FTIR, ^1^H-NMR, and ^13^C-NMR spectra, (Supplementary Materials, Figures S1–S16).

### 2.2. Biology

#### 2.2.1. Antiproliferative Activity

**Figure 2:**
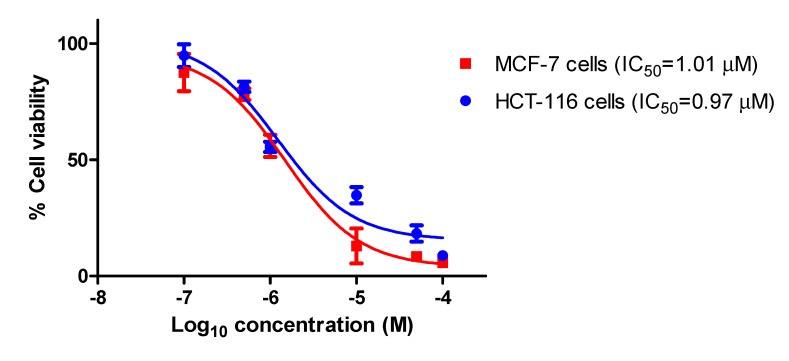
Antiproliferative effect of compound **11** in MCF-7 and HCT-116 cell lines. Cells were grown in 96-well plates and treated with compound **11** at 0.05–100 μM for 72 h. Cell viability was expressed as a percentage of vehicle control [ethanol 0.1% (*v*/*v*)] treated cells. The values represent the mean ± S.E.M. for three independent experiments performed in triplicate.

The antiproliferative activity of the synthesized *s*-triazine derivatives **7a–f, 8a–e, 9a–f,** and **10–12** were studied in human breast cancer (MCF-7) and colon carcinoma (HCT-116). Most of the *s*-triazine hydrazone derivatives affected the cell viability of the two cancer cell lines, as determined by the MTT cell viability assay. The results revealed that the most effective compound was **11** (compound containing two morpholine rings on the *s*-triazine core and pyridine at the hydrazone terminal) which affected the cell viability of the two cancer cell lines with IC_50_ values 1.0 µM and 0.98 µM in MCF-7 and HCT-116, respectively, Figure 2). Its analogs compound **10** (compound containing two-piperidine rings) and **12** (compound containing piperidine and morpholine ring) showed less potent activities as shown in Table 2. Therefore, the substituent on the *s*-triazine ring has a great effect on the activity of the tested compounds. Compounds containing two morpholine rings **8a–e** showed more potent antiproliferative activity in MCF-7 with IC_50_ values in the range 13.4–29.3 µM compared to their analogous containing two piperidine rings **7a–f** (IC_50_ values in the range 11.5–39.9 µM).

Compounds containing one morpholine and one piperidine ring **9a–f** showed better IC_50_ values in the range 10.4–22.2 µM. These data are in good agreement with our previously reported related *s*-triazine derivatives [24,27]. Interestingly, compounds containing two-piperidine rings **7a–f** affected the cell viability of the HCT-116 (IC_50_ values in the range 8.8–19.5 µM) more than their analogs **8a–e** (IC_50_ values in the range 18.3 > 50 µM) and **9a–f** (IC_50_ values in the range 22–42.2 µM) as shown in Table 2.

On the other hand, the substituent on the benzylidene moiety has also a great impact on the antiproliferative activity. As shown in Table 2, *p*-fluoro benzylidene derivatives **7e**, **8e**, and **9e** derivatives affected the cell viability of both cell lines in lower micromolar range compared to their analogs containing *p*-chloro (**7b**, **8b**, and **9b**) or *p*-bromo (**7c**, **8c**, and **9c**) benzylidene derivatives (Table 2). Thus, the presence of fluorine atoms significantly improves the pharmacological and physicochemical properties as the metabolic stability, lipophilicity as well as ligand binding [28,29], these results also agreed with the reported results by Bai et al. [22]. The replacement of fluorine atoms in compounds **7e**, **8e**, and **9e** with by hydroxy group as in compounds **7d**, **8d**, and **9d** showed also good antiproliferative activities against MCF-7 cell line with IC_50_ = 18.2, 14, and 10.4 µM, respectively (Table 2). These results agreed with the reported results which indicated that the phenolic hydroxyl group acts as a hydrogen donor which improves the anticancer activity [21,24].

## 3. Experimental Section

### 3.1. Materials and Methods

All solvents and reagents purchased from commercial suppliers and used without further purification. ^1^H NMR and ^13^C NMR spectra were recorded on a JEOL 400, 600 MHz spectrometer (JEOL, Ltd., Tokyo, Japan). Elemental analyses performed on Perkin-Elmer 2400 elemental analyzer (Perkin-Elmer, Inc.940 Winter Street, Waltham, MA, USA). Melting points were recorded on a Mel-Temp apparatus Sigma-Aldrich (Chemie GmbH, 82,024 Taufkirchen, Germany) in an open capillary and are uncorrected. Fourier transform infrared spectroscopy (FTIR) recorded on Shimadzu 8201 PC FTIR spectrophotometer (Shimadzu, Ltd., Kyoto, Japan).

### 3.2. General Method for the Synthesis of 1,3,5-Triazine-Hydrazone Derivatives

2-Hydrazino-4,6-disubstituted-1,3,5-triazine derivatives **2–4** were synthesized first following the synthetic strategy and methods reported by our group and others [20,21,22,23,24]. The target products were synthesized as follows: Aldehyde derivatives **5a–f** or **6** (10 mmol) in ethanol (10 mL) were added to a solution of **2–4** in ethanol (20 mL) containing 2–3 drops of acetic acid at room temperature. After complete addition the reaction mixture refluxed for 4–6 h, and the progress of the reaction was followed by thin-layer chromatography (TLC) using ethyl acetate–hexane 2:1. The reaction left to cool down to room temperature and the solid product separated by filtration, washed with cooled ethanol, and then dried at room temperature.

The characterization of compounds **8a–d** studied herein previously reported by our group [30]. The characterization of the remaining compounds described below.

#### 3.2.1. 2-(2-Benzylidenehydrazinyl)-4,6-di(piperidin-1-yl)-1,3,5-triazine, 7a

White solid in yield 82%; mp 240–242 °C; IR (KBr, cm^−1^): 3280 (NH), 1574 (C=N), 1504,1446 (C=C); ^1^H NMR (DMSO-*d_6_*): δ = 1.45 (s, 4H, 2CH_2_), 1.56 (s, 2H, CH_2_), 3.67 (s, 12H, 4 NCH_2_-, 2 OCH_2_−), 7.29–7.38 (m, 3H, 1H-3,4,5), 7.57 (d, 4H, *J* = 6.5 Hz, 2H-2,6), 8.02 (s, 1H, CH), 10.61 (s, 1H, NH) ppm; ^13^C NMR (DMSO-*d_6_*): δ = 24.4, 25.4, 43.4, 126.2, 128.7, 128.8, 135.2, 141.0, 164.2, 164.5 ppm. Anal. Calcd for C_20_H_27_N_7_ (365.48): C, 65.73; H, 7.45; N, 26.83. Found C, 65.87; H, 7.59; N, 26.98.

#### 3.2.2. 2-(2-(4-Chlorobenzylidene)hydrazinyl)-4,6-di(piperidin-1-yl)-1,3,5-triazine, 7b

White solid in yield 94%; mp 254–256 °C; IR (KBr, cm^−1^): 3284 (NH), 1579 (C=N), 1505,1444 (C=C); ^1^H NMR (DMSO-*d_6_*): δ = 1.47 (s, 8H, 4CH_2_), 1.59 (s, 4H, 2CH_2_), 3.71 (s, 12H, 4 NCH_2_-, 2 OCH_2_-), 7.45(d, 2H, *J* = 8.4, Ar), 7.62 (d, 2H, *J* = 8.8, Ar), 8.03 (s, 1H, CH), 10.73 (s, 1H, NH) ppm; ^13^C NMR (DMSO-*d_6_*): δ = 24.4, 25.4, 43.4, 127.8, 128.8, 133.1, 134.2, 139.6, 164.1, 164.5ppm. Anal. Calc. for C_20_H_26_ClN_7_ (399.92): C, 60.07; H, 6.55; N, 24.52. Found C, 60.23; H, 6.62; N, 24.71.

#### 3.2.3. 2-(2-(4-Bromobenzylidene)hydrazinyl)-4,6-di(piperidin-1-yl)-1,3,5-triazine, 7c

White solid in yield 95%; mp 253–255 °C; IR (KBr, cm^−1^): 3284 (NH), 1578 (C=N), 1503,1445 (C=C); ^1^H NMR (DMSO-*d_6_*): δ = 1.47 (s, 8H, 4CH_2_), 1.59 (s, 4H, 2CH_2_), 3.70 (s, 8H, 4CH_2_N), 7.54–7.60 (m, 4H, Ar), 8.02 (s, 1H, CH), 10.73 (s, 1H, NH) ppm; ^13^C NMR (DMSO-*d_6_*): δ = 24.4, 25.4, 43.5, 121.9, 128.1, 131.7, 134.5, 139.8, 164.2, 164.5ppm. Anal. Calc. for C_20_H_26_BrN_7_ (444.37): C, 54.06; H, 5.90; N, 22.06. Found C, 54.21; H, 5.98; N, 22.23.

#### 3.2.4. 4-((2-(4,6-Di(piperidin-1-yl)-1,3,5-triazin-2-yl)hydrazono)methyl)phenol, 7d

White solid in yield 97%; mp 150–152 °C; IR (KBr, cm^−1^): 3399(OH), 3145(NH), 1570(C=N), 1513, 1443(C=C);^1^H NMR (DMSO-*d_6_*): δ = 1.47 (s, 8H, 4CH_2_), 1.58 (s, 4H, 2CH_2_), 3.68 (s, 8H, 4CH_2_N), 6.78 (d, 2H, *J* = 8.0 Hz, Ar), 7.43 (d, 2H, *J* = 8.0 Hz, Ar), 7.94 (s, 1H, CH), 9.73 (s, 1H, OH), 10.40 (s, 1H, NH) ppm; ^13^C NMR (DMSO-*d_6_*): δ = 24.4, 25.5, 43.5, 115.6, 126.3, 127.9, 141.6, 158.4, 164.2, 164.6ppm. Anal. Calc. for C_20_H_27_N_7_O (381.47): C, 62.97; H, 7.13; N, 25.70. Found C, 63.12; H, 7.02; N, 25.89.

#### 3.2.5. 2-(2-(4-Fluorobenzylidene)hydrazinyl)-4,6-di(piperidin-1-yl)-1,3,5-triazine, 7e

White solid in yield 94%; mp 251–253 °C; IR (KBr, cm^−1^): 3222 (NH), 1564 (C=N), 1498, 1444 (C=C); ^1^H NMR (DMSO-*d_6_*): δ = 1.44 (s, 8H, 4CH_2_), 1.56 (s, 4H, 2CH_2_), 3.67 (s, 8H, 4CH_2_N), 7.20 (t, 2H, *J* = 9.0 Hz Ar), 7.62 (t.d, 2H, *J* = 7.3, 3.0 Hz, Ar), 8.01 (s, 1H, CH), 10.61 (s, 1H, NH) ppm; ^13^C NMR (DMSO-*d_6_*): δ = 24.6, 25.8, 44.0, 116.0, 128.5, 132.0, 140.2, 161.1, 164.2, 164.8ppm. Anal. Calc. for C_20_H_26_FN_7_ (383.47): C, 62.64; H, 6.83; N, 25.57. Found C, 62.88; H, 6.98; N, 25.34.

#### 3.2.6. 2-(2-(4-Methoxybenzylidene)hydrazinyl)-4,6-di(piperidin-1-yl)-1,3,5-triazine, 7f

White solid in yield 90%; mp 230–232 °C; IR (KBr, cm^−1^): 3286 (NH), 1578 (C=N), 1494,1448 (C=C); ^1^H NMR (DMSO-*d_6_*): δ = 1.47 (s, 8H, 4CH_2_), 1.59 (s, 4H, 2CH_2_), 3.69 (s, 8H, 4CH_2_N), 3.77 (s, 3H, OCH_3_), 6.96 (d, 2H, *J* = 8.8, Ar), 7.55 (d, 2H, *J* = 8.8, Ar), 7.99 (s, 1H, CH), 10.50 (s, 1H, NH) ppm; ^13^C NMR (DMSO-*d_6_*): δ = 24.4, 25.5, 43.4, 55.2, 114.2, 127.7, 127.9, 141.0, 159.9, 164.1, 164.4ppm. Anal. Calc. for C_21_H_29_N_7_O (395.50): C, 63.77; H, 7.39; N, 24.79. Found C, 63.91; H, 7.51; N, 24.96.

#### 3.2.7. 4,4’-(6-(2-(4-Fluorobenzylidene)hydrazinyl)-1,3,5-triazine-2,4-diyl)dimorpholine, 8e

White solid in yield 88%; mp 213–215 °C; IR (KBr, cm^−1^): 3237 (NH), 1564 (C=N), 1520, 1441 (C=C); ^1^H NMR (DMSO-*d_6_*): δ = 3.54–3.74 (m, 16H, 4 N-CH_2_-CH_2_-O), 7.20–7.23(m, 2H, Ar), 7.63–7.67 (m, 2H, Ar), 8.10 (s, 1H, CH), 10.77 (s, 1H, NH) ppm; ^13^C NMR (DMSO-*d_6_*): δ = 43.2, 66.0, 115.6, 128.4, 132.1, 140.6, 161.5, 164.3, 164.8 ppm. Anal. Calc. for: C_18_H_22_FN_7_O_2_ (387.41): C, 55.80; H, 5.72; N, 25.31. Found C, 56.01; H, 5.89; N, 25.95.

#### 3.2.8. 4-(4-(2-Benzylidenehydrazinyl)-6-(piperidin-1-yl)-1,3,5-triazin-2-yl)morpholine, 9a

White solid in yield 75%; mp 176–178 °C; IR (KBr, cm^−1^): 3224(NH), 1536(C=N), 1485,1441(C=C);^1^H NMR (DMSO-*d_6_*): δ = 1.44 (s, 4H, 2CH_2_), 1.56 (s, 2H, CH_2_), 3.53–3.67 (m, 12H, 4CH_2_N, 2 OCH_2_-), 7.28–7.38 (m, 3H, Ar), 7.59(d, 2H, *J* = 8.0, Ar), 8.03 (s, 1H, CH), 10.68 (s, 1H, NH) ppm; ^13^C NMR (DMSO-*d_6_*): δ 24.4, 25.4, 43.2, 43.5, 66.1, 126.3, 128.7, 128.9, 135.1, 141.3, 164.2, 164.9 ppm. Anal. Calc. for: C_19_H_25_N_7_O (367.45): C, 62.10; H, 6.86; N, 26.68. Found C, 62.29; H, 6.94; N, 26.79.

#### 3.2.9. 4-(4-(2-(4-Chlorobenzylidene)hydrazinyl)-6-(piperidin-1-yl)-1,3,5-triazin-2- yl)morpholine, 9b

White solid in yield 84%; mp 176–178 °C; IR (KBr, cm^−1^): 3229(NH), 1560(C=N), 1515,1483(C=C); ^1^H NMR (DMSO-*d_6_*): δ = 1.44 (s, 4H, 2CH_2_), 1.56 (s, 2H, CH_2_), 3.52–3.66 (m, 12H, 4CH_2_N, 2 OCH_2_-), 7.42 (d, 2H, *J* = 6.8, Ar), 7.60 (d, 2H, *J* = 6.5, Ar), 8.0 (s, 1H, CH), 10.76 (s, 1H, NH) ppm; ^13^C NMR (DMSO- *d_6_*): δ 24.4, 25.4, 43.2, 43.5, 66.0, 127.9, 128.8, 133.2, 134.1, 140.0, 164.2, 164.9 ppm. Anal. Calc. for: C_19_H_24_ClN_7_O (401.89): C, 56.78; H, 6.02; N, 24.40. Found C, 56.97; H, 6.18; N, 24.65.

#### 3.2.10. 4-(4-(2-(4-Bromobenzylidene)hydrazinyl)-6-(piperidin-1-yl)-1,3,5-triazin-2-yl)morpholine, 9c

White solid in yield 72%; mp 236–238 °C; IR (KBr, cm^−1^): 3270(NH), 1578(C=N), 1506,1483(C=C); ^1^H NMR (DMSO-*d_6_*): δ = 1.47 (s, 4H, 2CH_2_), 1.59 (s, 2H, CH_2_), 3.60–3.69 (m, 12H, 4CH_2_N, 2 OCH_2_-), 7.54–7.60 (m, 4H, Ar), 8.02 (s, 1H, CH), 10.79 (s, 1H, NH) ppm; ^13^C NMR (DMSO- *d_6_*): δ 24.4, 25.4, 43.3, 43.5, 66.0, 121.9, 128.1, 131.5, 134.2, 140.1, 164.2, 164.8 ppm. Anal. Calc. for: C_19_H_24_BrN_7_O (446.34): C, 51.13; H, 5.42; N, 21.97. Found C, 51.34; H, 5.58; N, 21.78.

#### 3.2.11. 4-((2-(4-Morpholino-6-(piperidin-1-yl)-1,3,5-triazin-2-yl)hydrazono)methyl)phenol, 9d

White solid in yield 76%; mp 156–158 °C; IR (KBr, cm^−1^): 3399 (OH), 3145(NH), 1570(C=N), 1515,1442(C=C); ^1^H NMR (DMSO-*d_6_*): δ = 1.50 (s, 4H, 2CH_2_), 1.62 (s, 2H, CH_2_), 3.59–3.71 (m, 12H, 4CH_2_N, 2 OCH_2_-), 6.81(d, 2H, *J* = 9.0, Ar), 7.47(d, 2H, *J* = 8.5, Ar), 7.99 (s, 1H, CH), 9.77 (s, 1H, OH), 10.51 (s, 1H, NH) ppm; 13C NMR (DMSO*-d_6_*): δ 24.4, 25.5, 43.2, 43.5, 66.1, 115.6, 126.2, 127.9, 141.9, 158.4, 164.1, 164.9 ppm; Anal. Calc. for: C_19_H_25_N_7_O_2_ (383.45): C, 59.51; H, 6.57; N, 25.57. Found C, 59.73; H, 6.71; N, 25.86.

#### 3.2.12. 4-(4-(2-(4-Fluorobenzylidene)hydrazinyl)-6-(piperidin-1-yl)-1,3,5-triazin-2-yl)morpholine, 9e

White solid in yield 90%; mp 240–242 °C; IR (KBr, cm^−1^): 3219(NH), 1563(C=N), 1518,1440(C=C); ^1^H NMR (DMSO-*d_6_*): δ = 1.47 (s, 4H, 2CH_2_), 1.59 (s, 2H, CH_2_), 3.60–3.69 (m, 12H, 4CH_2_N, 2 OCH_2_-), 7.23(t, 2H, *J* = 8.8, Ar), 7.66 (t.d, 2H, *J* = 7.0, 3.2 Hz, Ar), 8.05 (s, 1H, CH), 10.72 (s, 1H, NH) ppm; ^13^C NMR (DMSO-*d_6_*): δ 24.4, 25.4, 43.2, 43.4, 66.0, 116.0, 128.2, 132.8, 140.2, 161.1, 164.1, 164.9 ppm; Anal. Calc. for: C_19_H_24_FN_7_O (385.44): C, 59.21; H, 6.28; 25.44. Found C, 59.43; H, 6.40; 25.69.

#### 3.2.13. 4-(4-(2-(4-Methoxybenzylidene)hydrazinyl)-6-(piperidin-1-yl)-1,3,5-triazin-2-yl)morpholine, 9f

White solid in yield 76%; mp 175–177 °C; IR (KBr, cm^−1^): 3267(NH), 1556(C=N), 1516, 1486 (C=C); ^1^H NMR (DMSO-*d_6_*): δ = 1.44 (s, 4H, 2CH_2_), 1.56 (s, 2H, CH_2_), 3.54–3.66 (m, 12H, 4CH_2_N, 2 OCH_2_-), 3.74 (s, 3H, OCH_3_), 6.93(d, 2H, *J* = 9.5, Ar), 7.53(d, 2H, *J* = 9.5, Ar), 8.0 (s, 1H, CH), 10.54 (s, 1H, NH) ppm; ^13^C NMR (DMSO-*d_6_*): δ 24.4, 25.4, 43.2, 43.4, 55.2, 66.1, 114.2, 127.7, 127.8, 141.3, 159.9, 164.3, 164.9 ppm. Anal. Calc. for: C_20_H_27_N_7_O_2_ (397.47): C, 60.44; H, 6.85; N, 24.67. Found C, 60.68; H, 6.96; N, 24.88.

#### 3.2.14. 2,4-Di(piperidin-1-yl)-6-(2-(pyridin-2-ylmethylene)hydrazinyl)-1,3,5-triazine, 10

White solid in yield 75%; mp 196–198 °C; IR (KBr, cm^−1^): 3223 (NH), 1577(C=N), 1514, 1440 (C=C); ^1^H NMR (DMSO-*d_6_*): δ = 1.48 (s, 8H, 4CH_2_), 1.60 (s, 4H, 2CH_2_), 3.71 (s, 8H, 4CH_2_N), 7.29–7.32(m, 2H, Ar), 7.80–7.88 (m, 2H, Ar), 8.10 (s, 1H, CH), 8.53 (d, 1H, *J* = 5.2 Hz, Ar), 10.90 (s, 1H, NH) ppm; ^13^C NMR (DMSO-d_6_): δ = 24.4, 25.4, 43.5, 119.0, 123.1, 136.5, 141.5, 149.3,154.0, 164.1, 164.3ppm; Anal. Calc. for C_19_H_26_N_8_ (366.46): C, 62.27; H, 7.15; N, 30.58. Found C, 62.45; H, 7.31; N, 30.81.

#### 3.2.15. 4,4’-(6-(2-(Pyridin-2-ylmethylene)hydrazinyl)-1,3,5-triazine-2,4-diyl)dimorpholine, 11

White solid in yield 92%; mp 203–205 °C; IR (KBr, cm^−1^): 3219(NH), 1568(C=N), 1516, 1435(C=C); ^1^H NMR (DMSO-*d_6_*): δ = 3.53–3.67 (m, 16H, 4 N-CH_2_-CH_2_-O), 7.44–7.46(m, 2H, Ar), 7.64 (d, 2H, *J* = 7.5 Hz, Ar), 8.00 (s, 1H, CH), 8.71 (d, 1H, *J* = 4.5 Hz, Ar), 11.00 (s, 1H, NH) ppm; ^13^C NMR (DMSO-*d_6_*): δ = 43.2, 66.0, 123.9, 125.6, 133.6, 138.1, 148.2, 152.5, 164.5, 164.8ppm; Anal. Calc. for C_17_H_22_N_8_O_2_ (370.41): C, 55.12; H, 5.99; N, 30.25. Found C, 55.36; H, 5.81; N, 30.04.

#### 3.2.16. 4-(4-(Piperidin-1-yl)-6-(2-(pyridin-2-ylmethylene)hydrazinyl)-1,3,5-triazin-2-yl)morpholine, 12

White solid in yield 72%; mp 236–238 °C; IR (KBr, cm-1): 3223(NH), 1572(C=N), 1514,1439(C=C); ^1^H NMR (DMSO-d_6_): δ = 1.45(s, 4H, 2CH_2_), 1.57 (s, 2H, CH_2_), 3.54–3.67 (m, 12H, 4CH_2_N, 2 OCH_2_-),7.27–7.29 (m, 2H, Ar), 7.76–7.85 (m, 2H, Ar), 8.08 (s, 1H, CH), 8.50 (d, 1H, J = 5.2 Hz, Ar), 10.94 (s, 1H, NH) ppm; ^13^C NMR (DMSO-d_6_): δ 24.3, 25.4, 43.2, 43.5, 66.0, 119.1, 123.3, 136.6, 141.9, 149.2,154.0, 164.2, 164.9 ppm; Anal. Calc. for: C_18_H_24_N_8_O (368.44): C, 58.68; H, 6.57; N, 30.41. Found C, 58.91; H, 6.66; N, 30.20.

### 3.3. Biology

#### 3.3.1. In Vitro Antiproliferative Assay

The target products **7a–f**, **8a–e**, **9a–f**, **10–12** were evaluated for antiproliferative activity using the MTT (Tetrazolium salt (3-(4,5-dimethylthiazol-2-yl)-2,5-diphenyltetrazolium bromide) viability assay in human breast cancer (MCF-7) and colon carcinoma (HCT-116) cell lines. Cells were seeded in triplicate in 96-well plates at a density of 10 × 10^3^ cells/mL in a total volume of 200 µL per well. 0.1% of DMSO used as vehicle control. Each well was treated with 2 µL tested compounds, which had been pre-prepared as stock solutions in ethanol to furnish the concentration range of study, 10 nM to 100 µM, and re-incubated for a further 72 h. The culture medium was then removed and the cells washed with 100 µL phosphate-buffered saline (PBS) and 50 µL MTT added, to reach a final concentration of 1 mg/mL MTT added. Cells incubated for 2 h in darkness at 37 °C. At this point, solubilization was begun through the addition of 200 mL DMSO and the cells maintained at room temperature in darkness for 20 min to ensure thorough color diffusion before reading the absorbance. Plates were incubated for 72 h at 37 °C + 5% CO_2_. The MTT (5 mg/mL in PBS) was added and incubated for another 4 h, the optical density was detected with a microplate reader at 570 nm. Results expressed as percentage viability relative to vehicle control (100%). Dose–response curves plotted and IC_50_ values (concentration of drug resulting in 50% reduction in cell survival) were obtained using the commercial software package Prism (GraphPad Software, Inc., La Jolla, CA, USA). All the experiments were repeated in at least three independent experiments.

## 4. Conclusion

The *s*-triazine hydrazone derivatives reported herein were evaluated for their inhibitory effect of the growth of two human cancer cell lines; breast cancer MCF-7 and colon cancer HCT-116 using MTT assay. Compound **11** which containing two morpholine rings on the *s*-triazine core and pyridine at the hydrazone terminal showed the greatest antiproliferative activity amongst all the tested compounds (IC_50_ = 1.0 µM and 0.98 µM in MCF-7 and HCT-116, respectively). The substituent on the *s-*triazine ring has a great effect on the activity of the tested compounds. This was observed with compound **11** vs. **10** (IC_50_ = 32.8 µM in MCF-7 and >50 µM in HCT-116) and **12** (IC_50_ = 17.7 µM in MCF-7 and 30.4 µM in HCT-116). This also was noticed in the series of compounds containing two morpholine rings **8a–e** which showed more potent antiproliferative activity in MCF-7 (IC_50_ values in the range 13.4–29.3 µM) compared to their analogous containing two-piperidine rings **7a–f** (IC_50_ values in the range 11.5–39.9 µM). While compounds containing one morpholine and one piperidine rings **9a–f** showed better IC_50_ values in the range 10.4–22.2 µM, these results agreed with the reported in the literature [24,27]. Interestingly, compounds containing two-piperidine rings **7a–f** showed more potent activity in HCT-116 cell line (IC_50_ values in the range 8.8–19.5 µM) compared with their analogs **8a–e** (IC_50_ values in the range 18.3 > 50 µM) and **9a–f** (IC_50_ values in the range 22–42.2 µM).

The substituent on the benzylidene moiety also affected the antiproliferative activity, where derivatives with *p*-fluoro derivatives (**7e**, **8e**, and **8e**) exhibited more potent activities in both tested cell lines (IC_50_ = 11.5, 13.4, 13.9 µM in MCF-7 and 14, 18.3, 22 µM in HCT-116, respectively) than their analogs *p*-chloro (**7b**, **8b**, and **9b**) and *p*-bromo (**7c**, **8c**, and **9c**) derivatives. Replacement of the fluorine atom by hydroxy group showed better anticancer activities compared to its analogous methoxy group.

Taken together, these results efforts on the synthesis of more new series based on *s*-triazine hydrazone derivatives with different groups in progress in our lab, proving the antiproliferative activity and possible mechanism of action of s-triazine hydrazone derivatives which might be of special interest in medicinal chemistry.

## Figures and Tables

**Table 1:** Structure of compounds **7a–f**, **8a–e**, **9a–f**, and **10–12** as shown in Scheme 1.

Cpd No.	R_1_	R_2_	X	Cpd No.	R_1_	R_2_	X
**7a**	piperidine	piperidine	H	**9a**	morpholine	piperidine	H
**7b**	piperidine	piperidine	Cl	**9b**	morpholine	piperidine	Cl
**7c**	piperidine	piperidine	Br	**9c**	morpholine	piperidine	Br
**7d**	piperidine	piperidine	OH	**9d**	morpholine	piperidine	OH
**7e**	piperidine	piperidine	F	**9e**	morpholine	piperidine	F
**7f**	piperidine	piperidine	OCH_3_	**9f**	morpholine	piperidine	OCH_3_
**8a**	morpholine	morpholine	H	**10**	piperidine	piperidine	
**8b**	morpholine	morpholine	Cl	**11**	morpholine	morpholine	
**8c**	morpholine	morpholine	Br	**12**	morpholine	piperidine	
**8d**	morpholine	morpholine	OH				
**8e**	morpholine	morpholine	F				

**Table 2:** Antiproliferative activities of compounds **7a–f**, **8a–e**, **9a–f**, and **10**–**12** in MCF-7 and HCT-116 cell lines.

Compound No.	IC_50_ (μM) MCF-7	IC_50_ (μM) HCT-116
**7a**	17.5 ± 5.9	12.6 ± 4.6
**7b**	23.5 ± 8.0	10.8 ± 3.5
**7c**	39.8 ± 12.9	10.9 ± 3.6
**7d**	18.2 ± 5.8	19.5 ± 6.2
**7e**	11.5 ± 3.3	14.0 ± 4.4
**7f**	39.9 ± 12.7	8.8 ± 2.5
**8a**	29.3 ± 9.3	>50
**8b**	17.6 ± 5.7	19.2 ± 6.2
**8c**	23.2 ± 7.3	38.7 ± 12.4
**8d**	14.0 ± 4.8	29.9 ± 9.5
**8e**	13.4 ± 4.0	18.3 ± 5.7
**9a**	22.2 ± 7.9	44.2 ± 14.4
**9b**	21.9 ± 6.0	30.0 ± 9.6
**9c**	22.5 ± 3.9	28.2 ± 9.0
**9d**	10.4 ± 3.1	25.4 ± 8.2
**9e**	13.9 ± 4.7	22.0 ± 7.1
**9f**	14.2 ± 4.5	23.4 ± 7.4
**10**	32.8 ± 10.4	>50
**11**	1.0 ± 0.3	0.98 ± 0.3
**12**	17.7 ± 5.5	30.4 ± 9.9

## Supplementary Materials

The following are available online, Figure S1: 1H-NMR and 13C-NMR for compound 7a, Figure S2: 1H-NMR and 13C-NMR for compound 7b, Figure S3: 1H-NMR and 13C-NMR for compound 7c, Figure S4: 1H-NMR and 13C-NMR for compound 7d, Figure S5: 1H-NMR and 13C-NMR for compound 7e, Figure S6: 1H-NMR and 13C-NMR for compound 7f, Figure S7: 1H-NMR and 13C-NMR for compound 8e, Figure S8: 1H-NMR and 13C-NMR for compound 9a, Figure S9: 1H-NMR and 13C-NMR for compound 9b, Figure S10: 1H-NMR and 13C-NMR for compound 9c, Figure S11: 1H-NMR and 13C-NMR for compound 9d, Figure S12: 1H-NMR and 13C-NMR for compound 9e, Figure S13: 1H-NMR and 13C-NMR for compound 9f, Figure S14: 1H-NMR and 13C-NMR for compound 10, Figure S15: 1H-NMR and 13C-NMR for compound 11, Figure S16: 1H-NMR and ^13^C-NMR for compound 12.
