# Supplementary material for: Synthesis and Characterization of New Series of 1,3-5-Triazine Hydrazone Derivatives with Promising Antiproliferative Activity
Source: Molecules. 2020 Jun 11;25(11):2708. doi: 10.3390/molecules25112708 (PMC7321239; doi:10.3390/molecules25112708)
Supplement: Supplementary file 1 [file molecules-25-02708-s001.pdf]

molecules

Article

Synthesis and Characterization of New Series of 1,3-5-Triazine Hydrazone Derivatives with Promising Antiproliferative Activity

Hessa H. Al Rasheed [1,*], Azizah M. Malebari [2] and Kholood A. Dahlous [1], Ayman El-Faham [1,3,*]

[1] Department of Chemistry, College of Science, King Saud University P.O. Box 2455, Riyadh 11451, Saudi Arabia; kdahloos@KSU.EDU.SA

[2] Department of Pharmaceutical Chemistry, College of Pharmacy, King Abdulaziz University, P.O.Box : 80260, Jeddah 21589, Saudi Arabia; amelibary@kau.edu.sa

[3] Chemistry Department, Faculty of Science, Alexandria University, P.O. Box 426, Ibrahimia, Alexandria 12321, Egypt

* Correspondence: halbahli@ksu.edu.sa (H.H.A.R.); aelfaham@ksu.edu.sa or aymanel_faham@hotmail.com (A.E.-F.); Tel.: +96-61-1467-3195 (A.E-F.)

Table of Content

^{1}H-NMR and ^{13}C-NMR for compound **7a**	**Figure S1**
^{1}H-NMR and ^{13}C-NMR for compound **7b**	**Figure S2**
^{1}H-NMR and ^{13}C-NMR for compound **7c**	**Figure S3**
^{1}H-NMR and ^{13}C-NMR for compound **7d**	**Figure S4**
^{1}H-NMR and ^{13}C-NMR for compound **7e**	**Figure S5**
^{1}H-NMR and ^{13}C-NMR for compound **7f**	**Figure S6**
^{1}H-NMR and ^{13}C-NMR for compound **8a**	**Figure S7**
^{1}H-NMR and ^{13}C-NMR for compound **8b**	**Figure S8**
^{1}H-NMR and ^{13}C-NMR for compound **8c**	**Figure S9**
^{1}H-NMR and ^{13}C-NMR for compound **8d**	**Figure S10**
^{1}H-NMR and ^{13}C-NMR for compound **8e**	**Figure S11**
^{1}H-NMR and ^{13}C-NMR for compound **9a**	**Figure S12**
^{1}H-NMR and ^{13}C-NMR for compound **9b**	**Figure S13**
^{1}H-NMR and ^{13}C-NMR for compound **9c**	**Figure S14**
^{1}H-NMR and ^{13}C-NMR for compound **9d**	**Figure S15**
^{1}H-NMR and ^{13}C-NMR for compound **9e**	**Figure S16**
^{1}H-NMR and ^{13}C-NMR for compound **9f**	**Figure S17**
^{1}H-NMR and ^{13}C-NMR for compound **10**	**Figure S18**
^{1}H-NMR and ^{13}C-NMR for compound **11**	**Figure S19**
^{1}H-NMR and ^{13}C-NMR for compound **12**	**Figure S20**

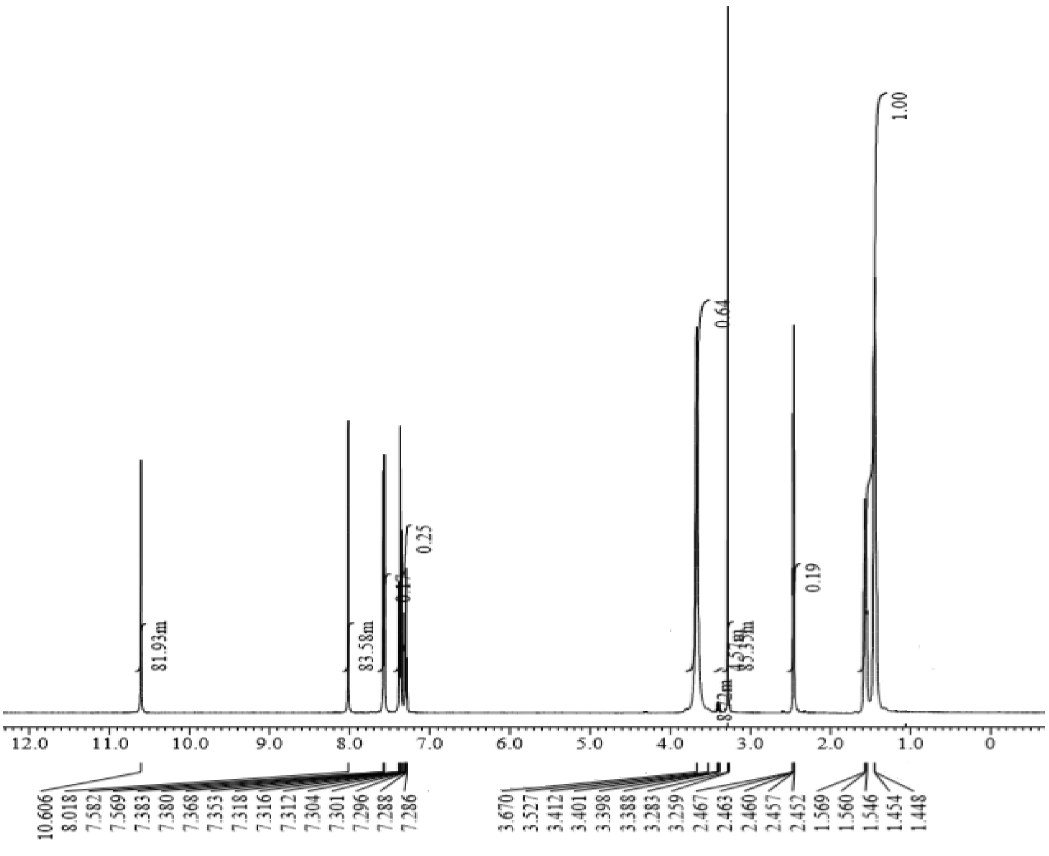

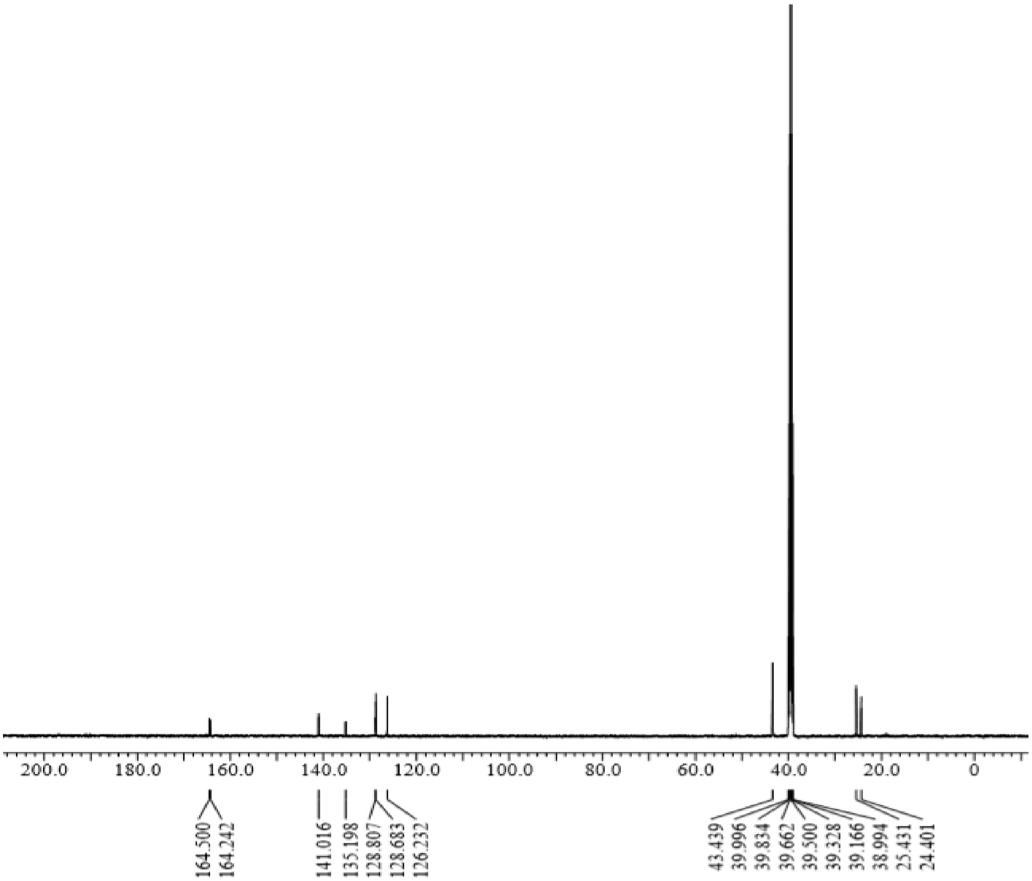

Figure S1. ^{1}H-NMR and ^{13}C-NMR for compound 7a.

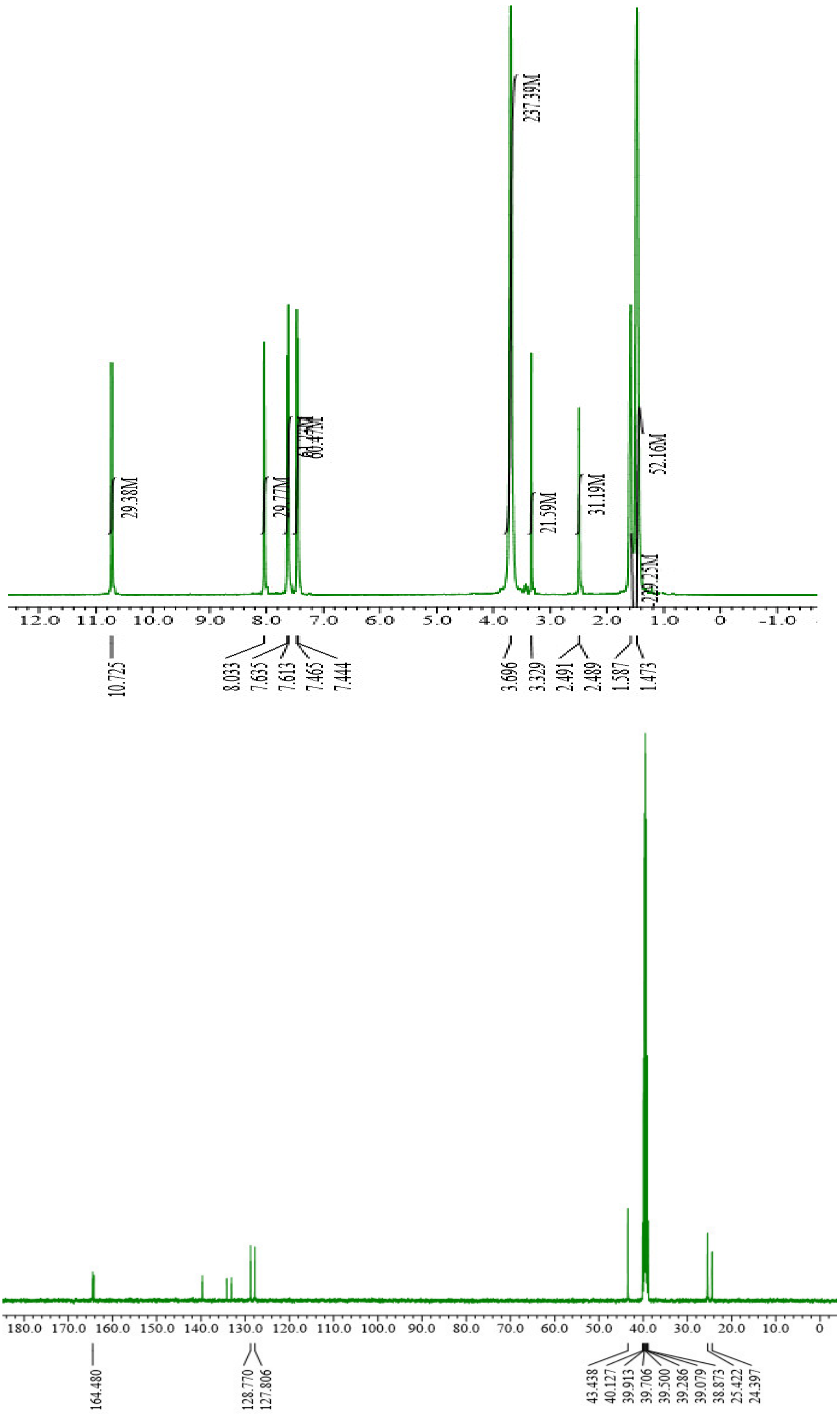

Figure S2. ^{1}H-NMR and ^{13}C-NMR for compound 7b.

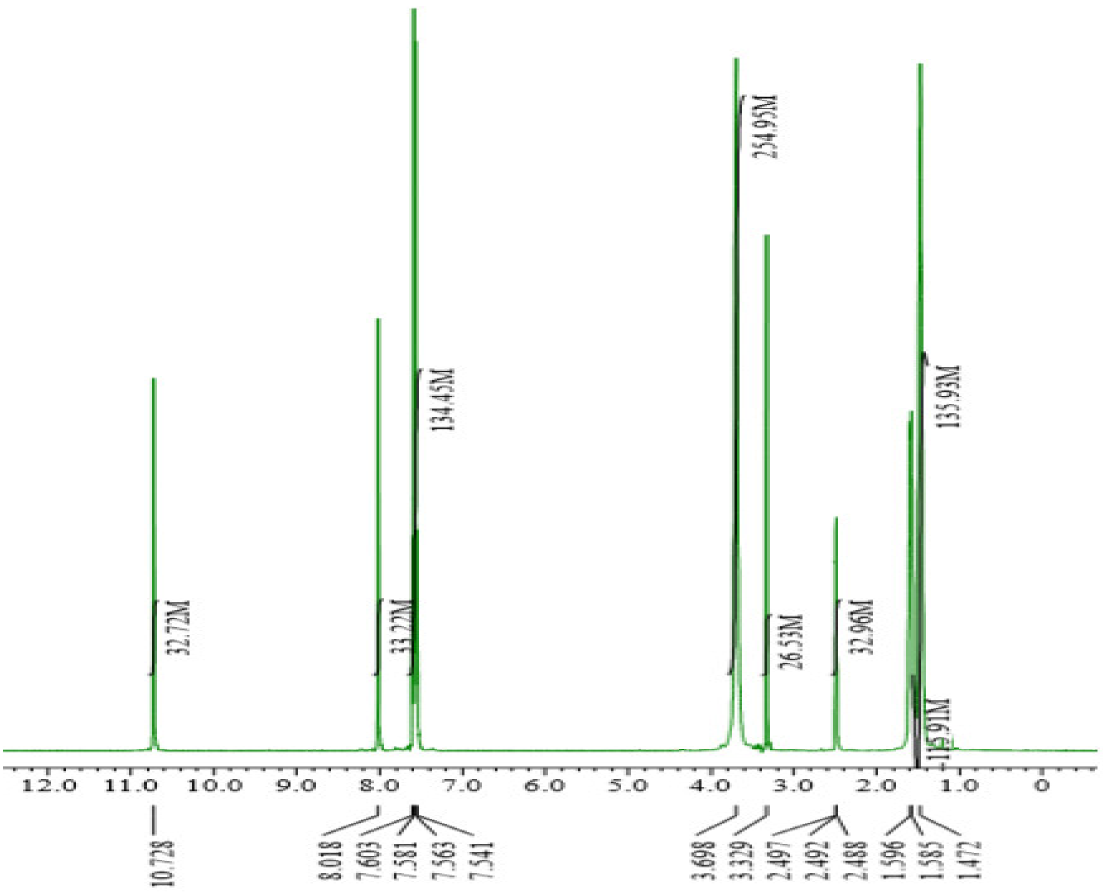

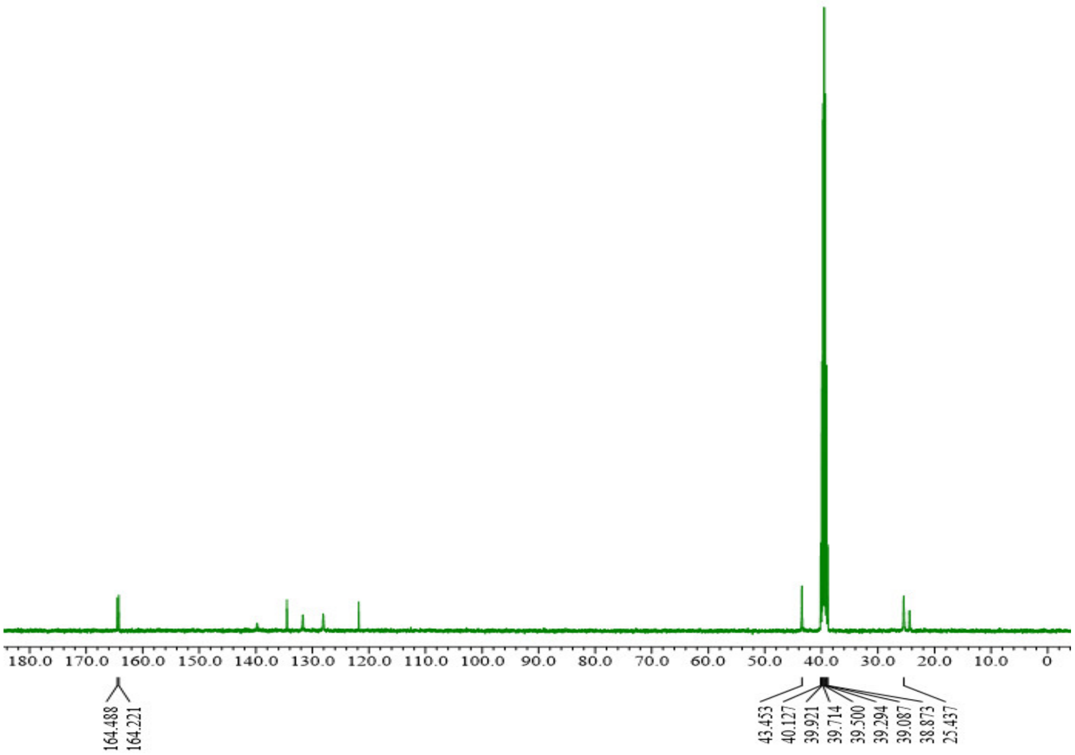

Figure S3. ^{1}H-NMR and ^{13}C-NMR for compound **7c.**

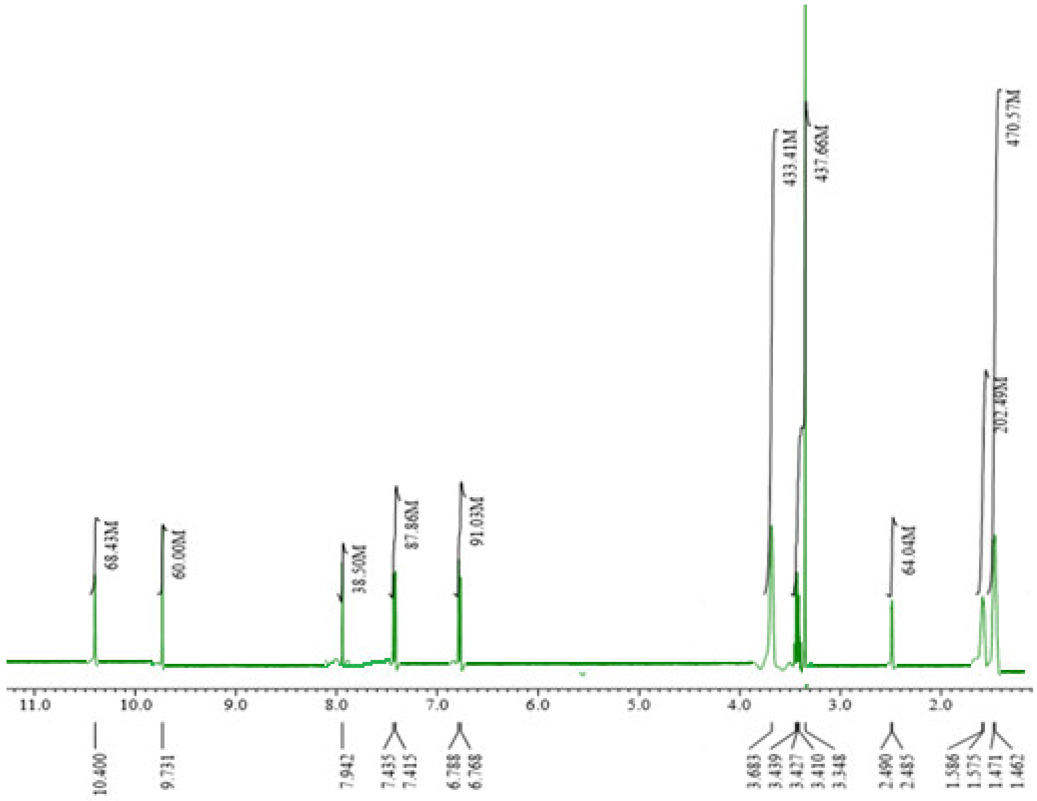

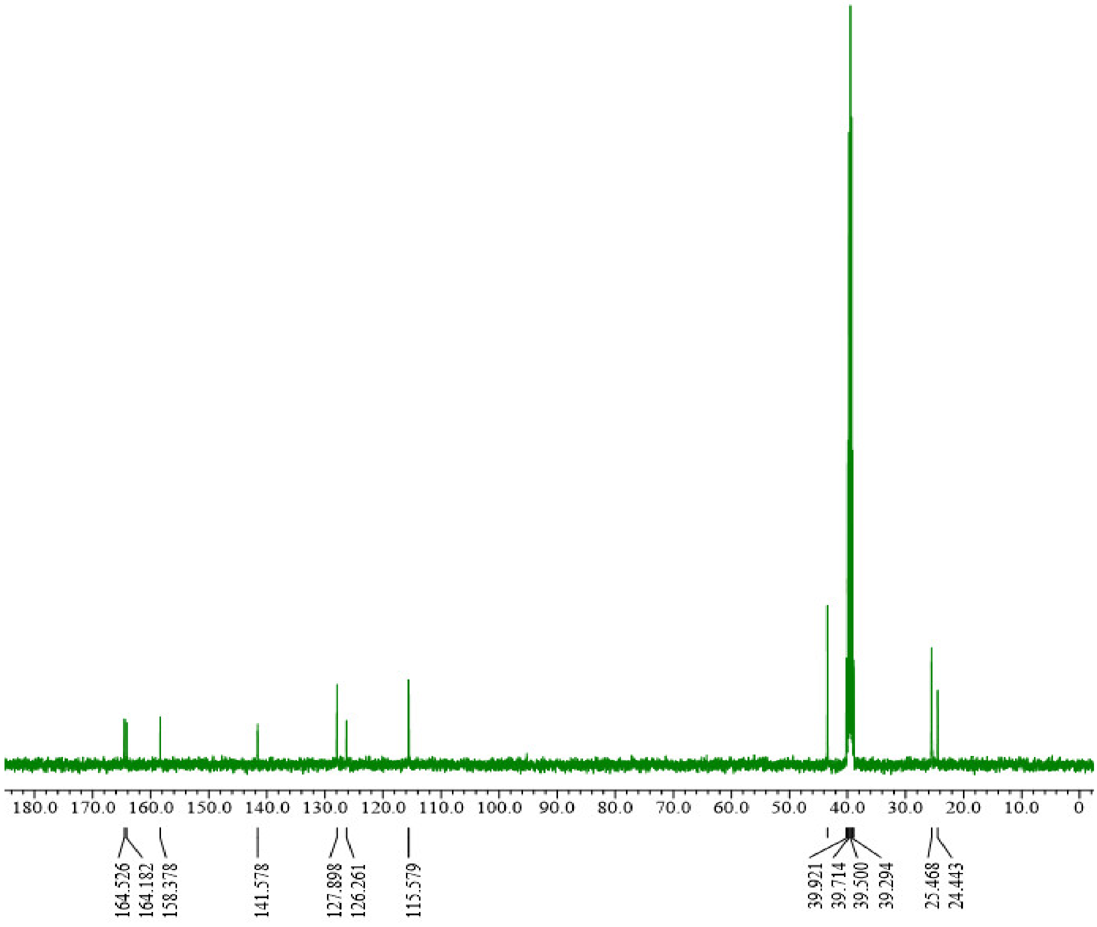

Figure S4. ^{1}H-NMR and ^{13}C-NMR for compound **7d.**

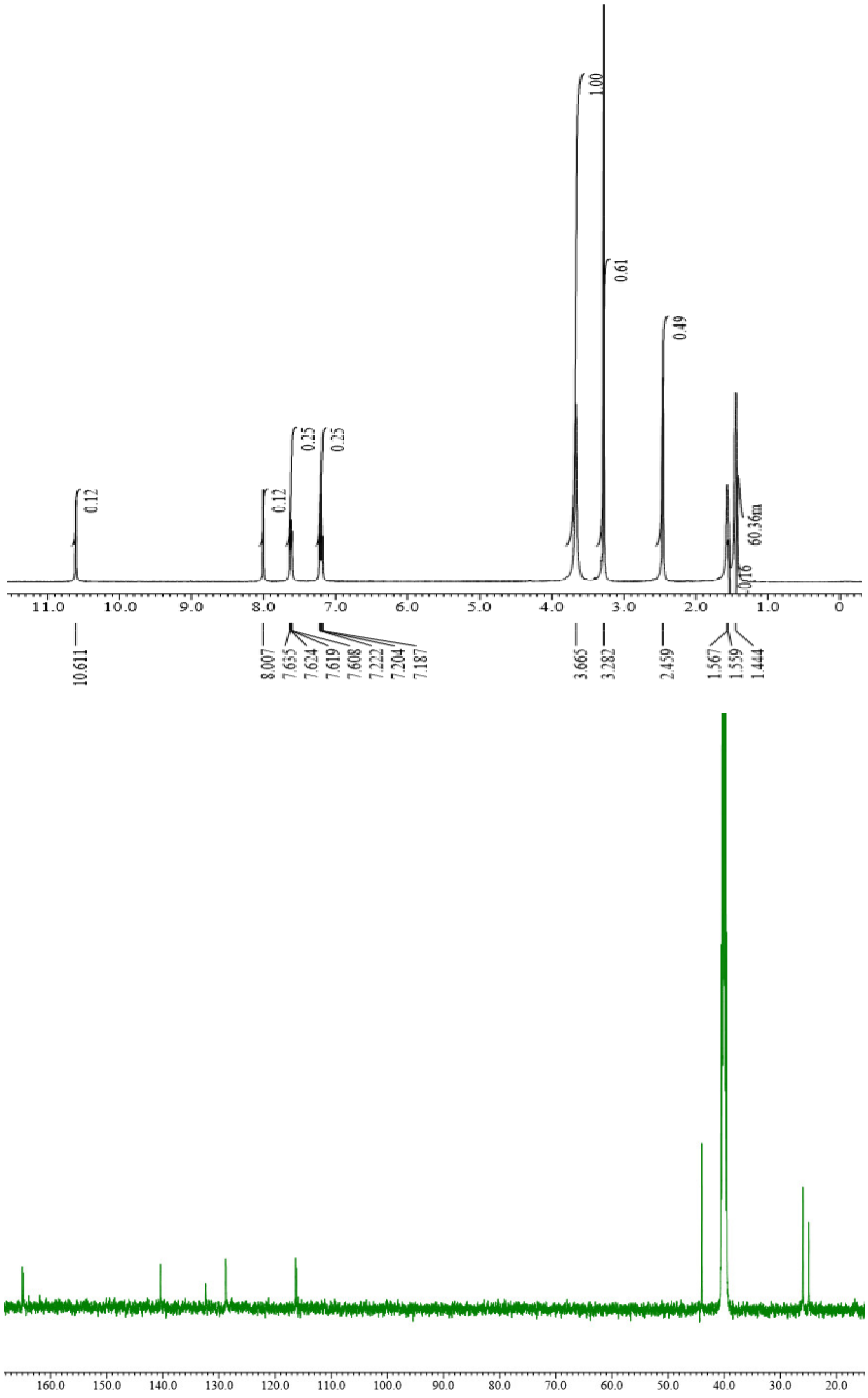

Figure S5. ^{1}H-NMR and ^{13}C-NMR for compound **7e.**

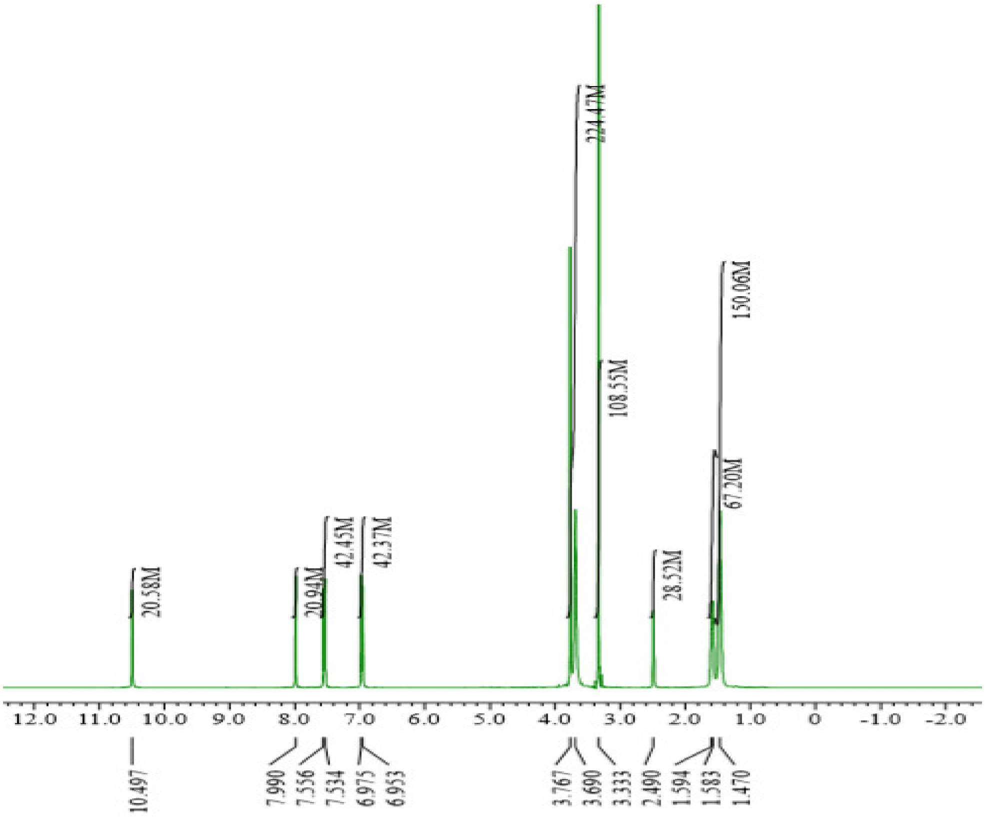

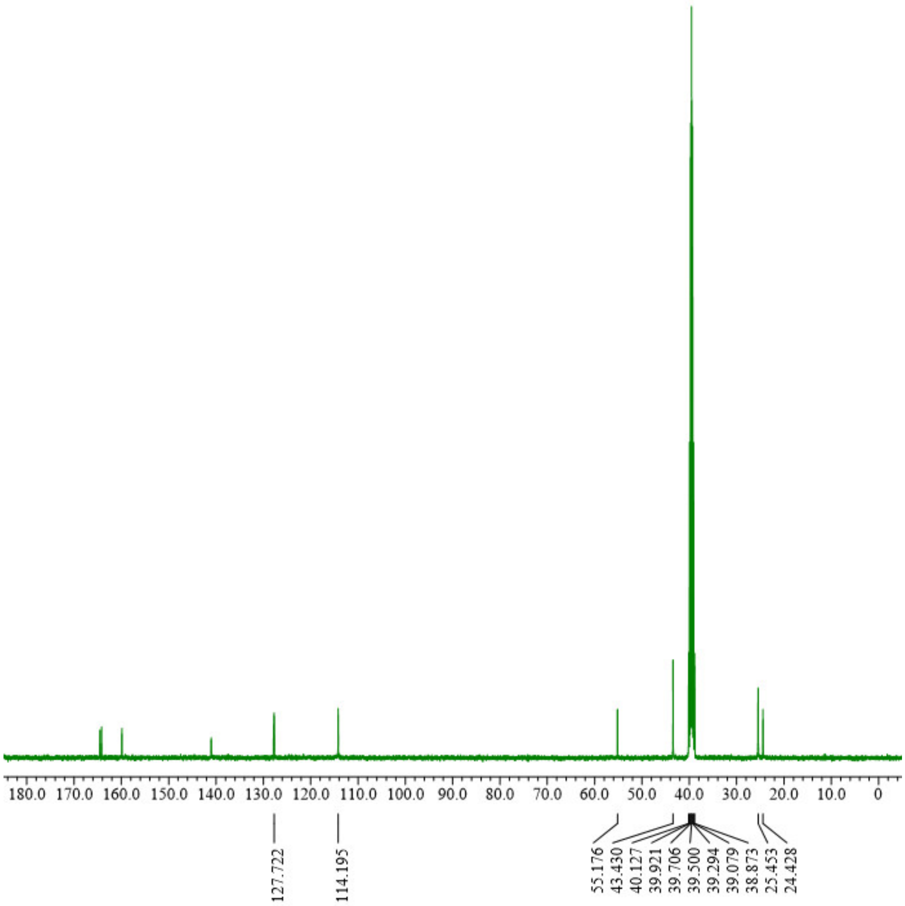

Figure S6. ^{1}H-NMR and ^{13}C-NMR for compound **7f.**

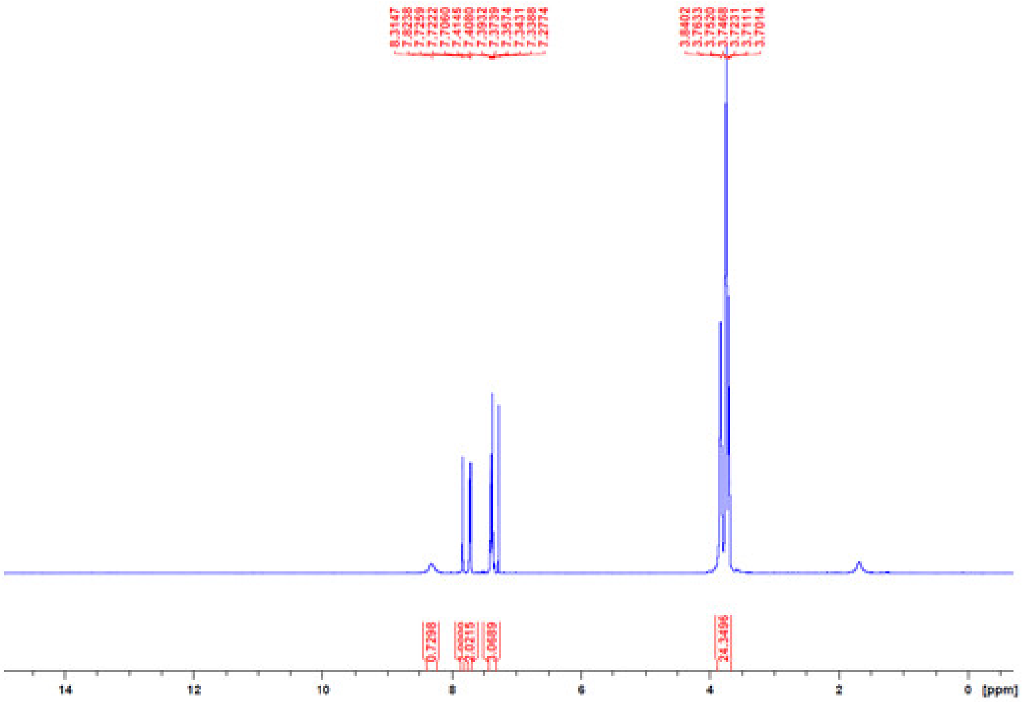

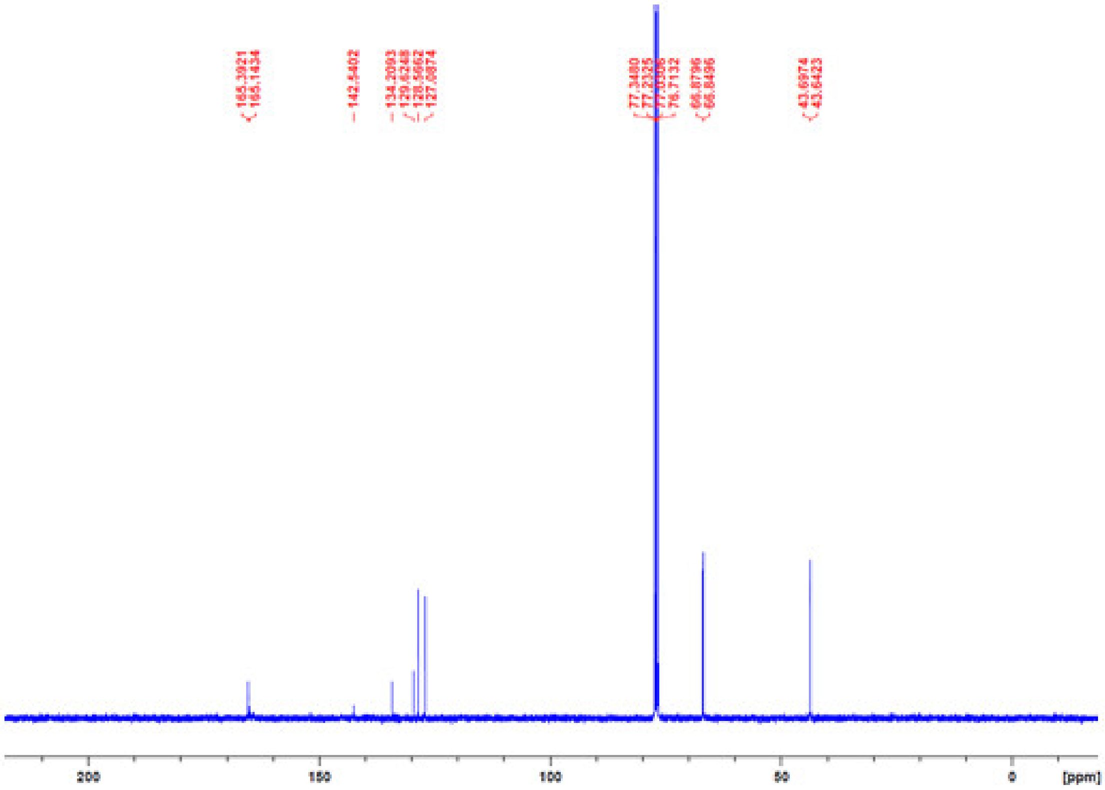

Figure S7. ^{1}H-NMR and ^{13}C-NMR for compound **8a.**

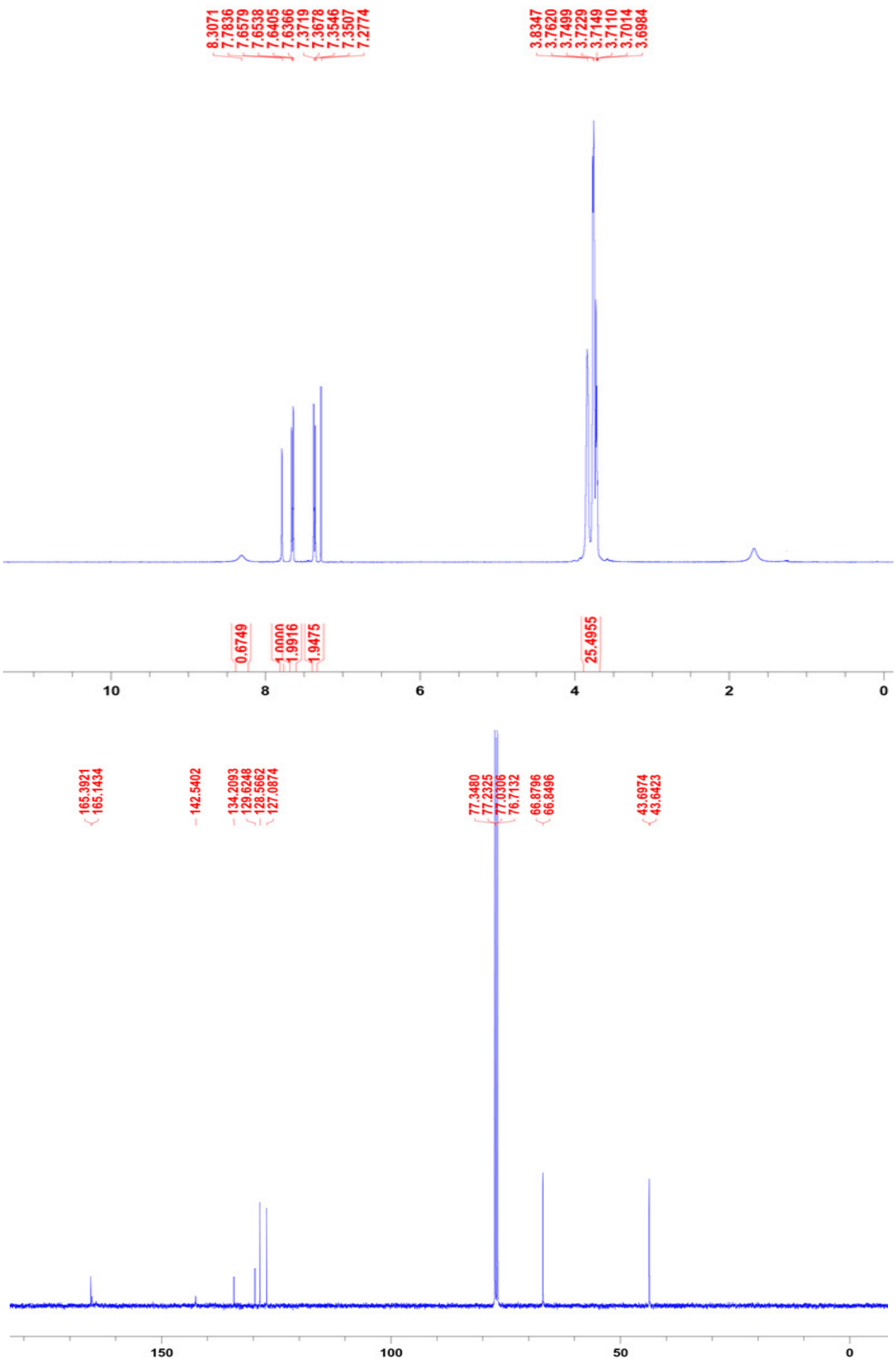

Figure S8. ^{1}H-NMR and ^{13}C-NMR for compound **8b.**

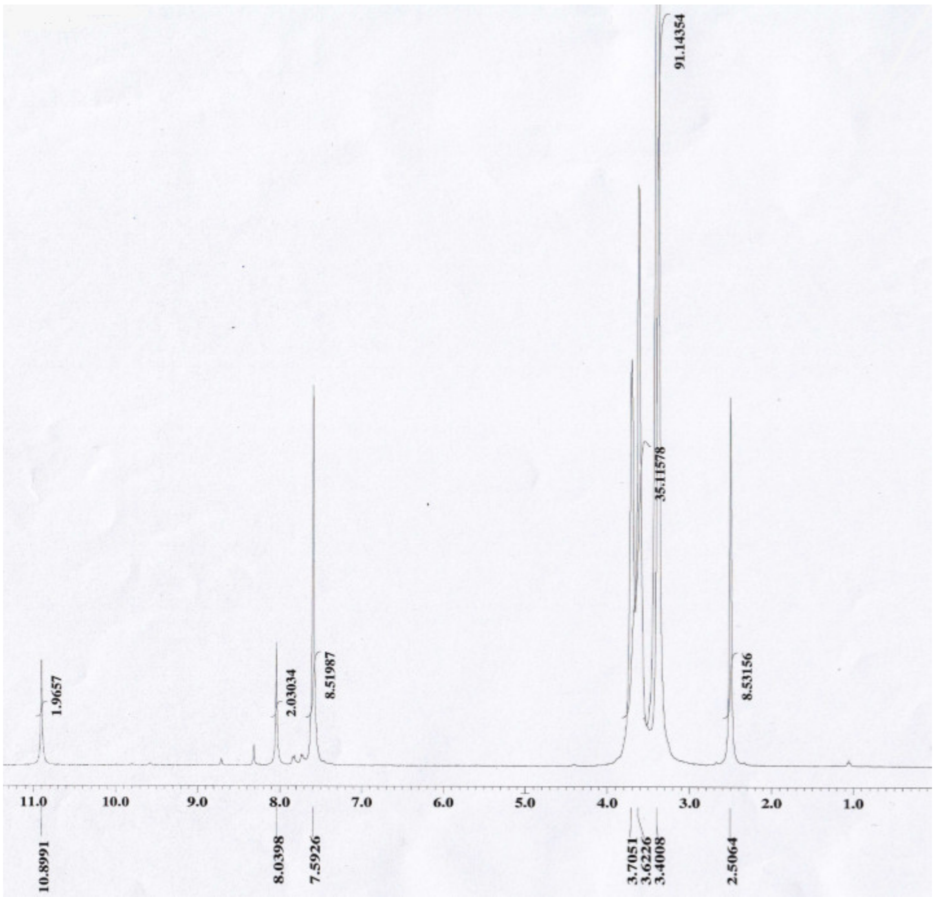

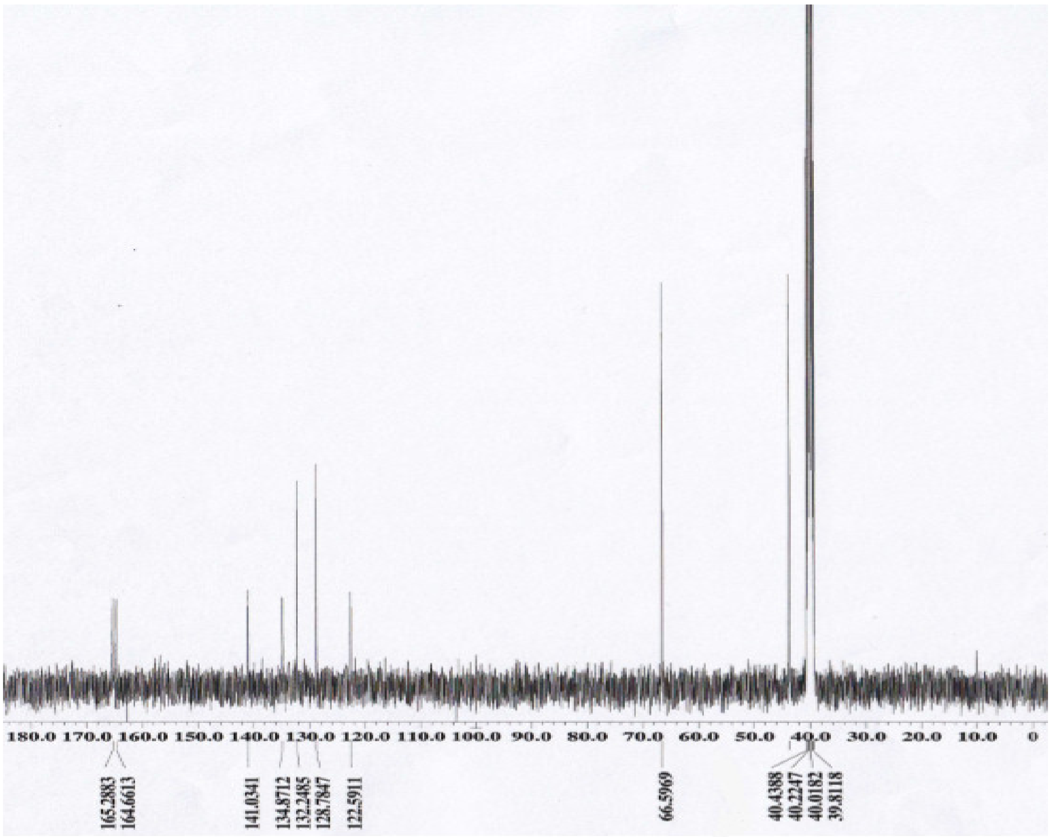

Figure S9. ¹H-NMR and ¹³C-NMR for compound **8c.**

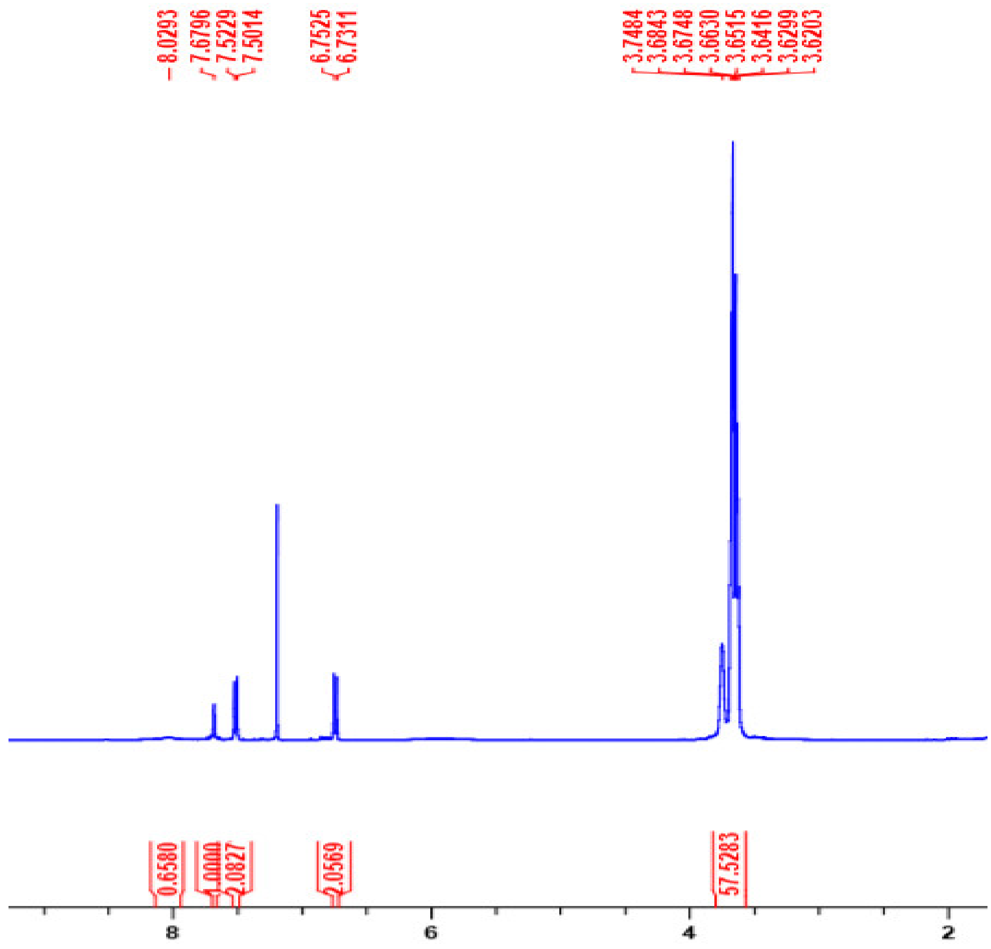

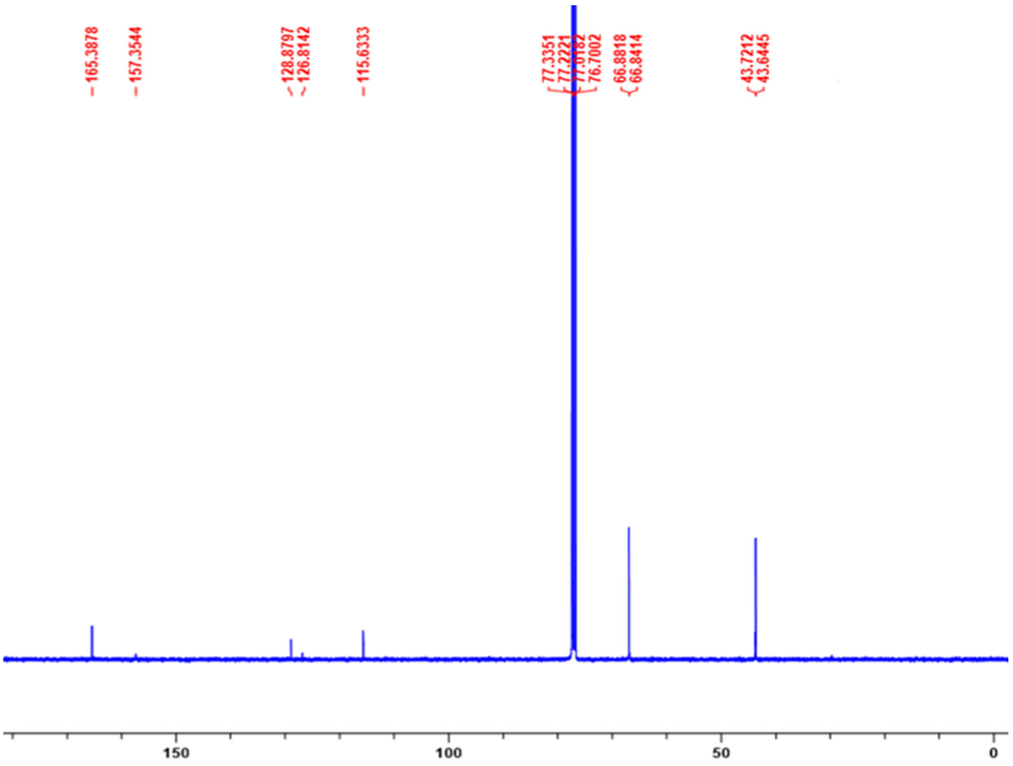

Figure S10. ^{1}H-NMR and ^{13}C-NMR for compound **8d.**

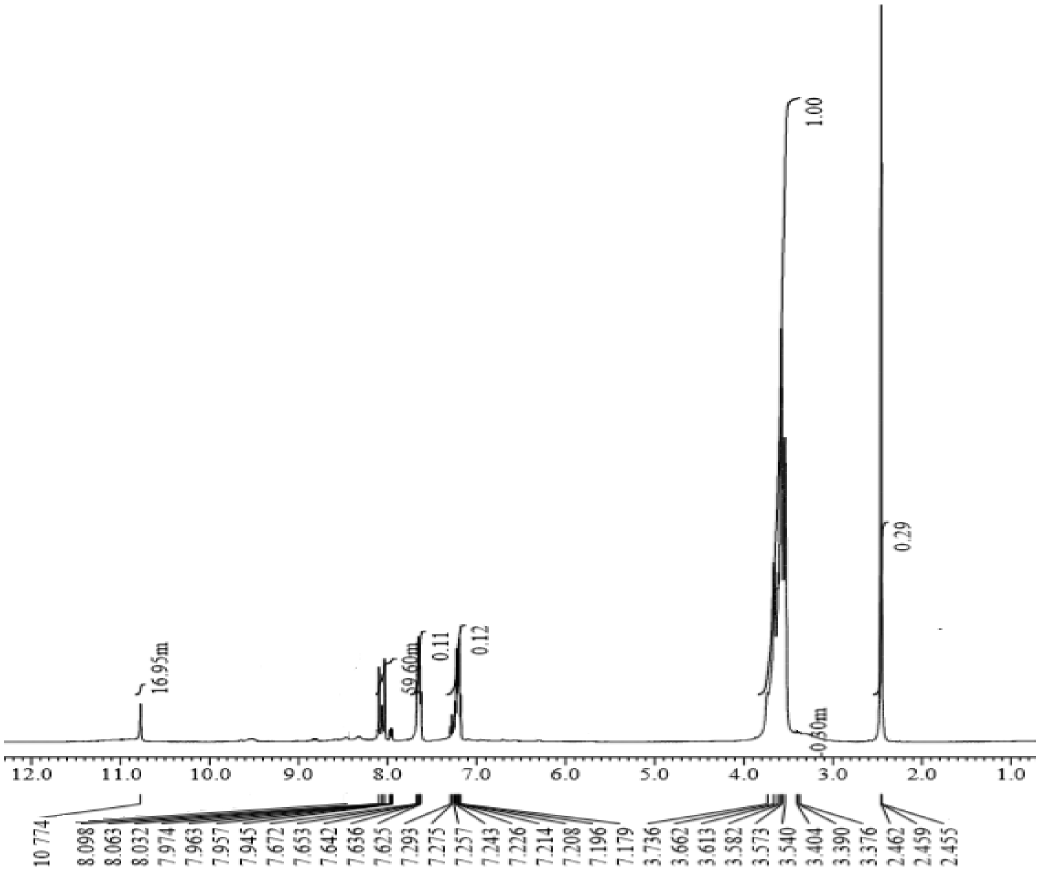

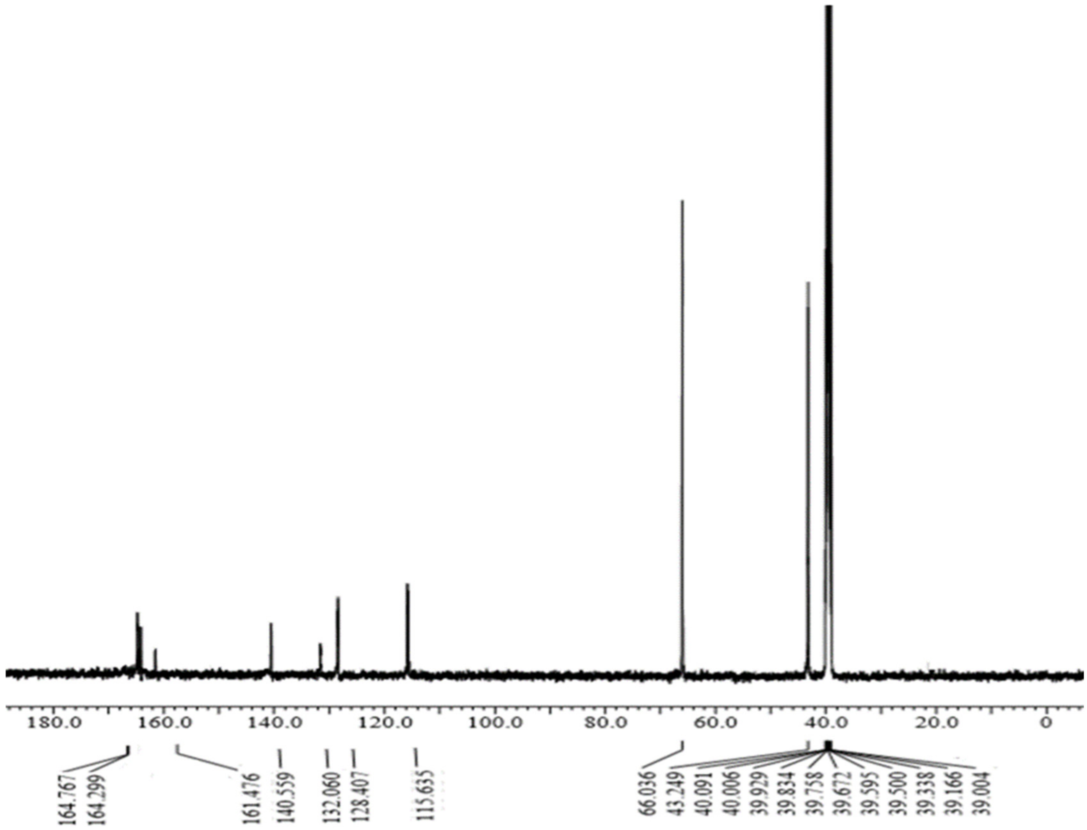

Figure S11. ^{1}H-NMR and ^{13}C-NMR for compound **8e.**

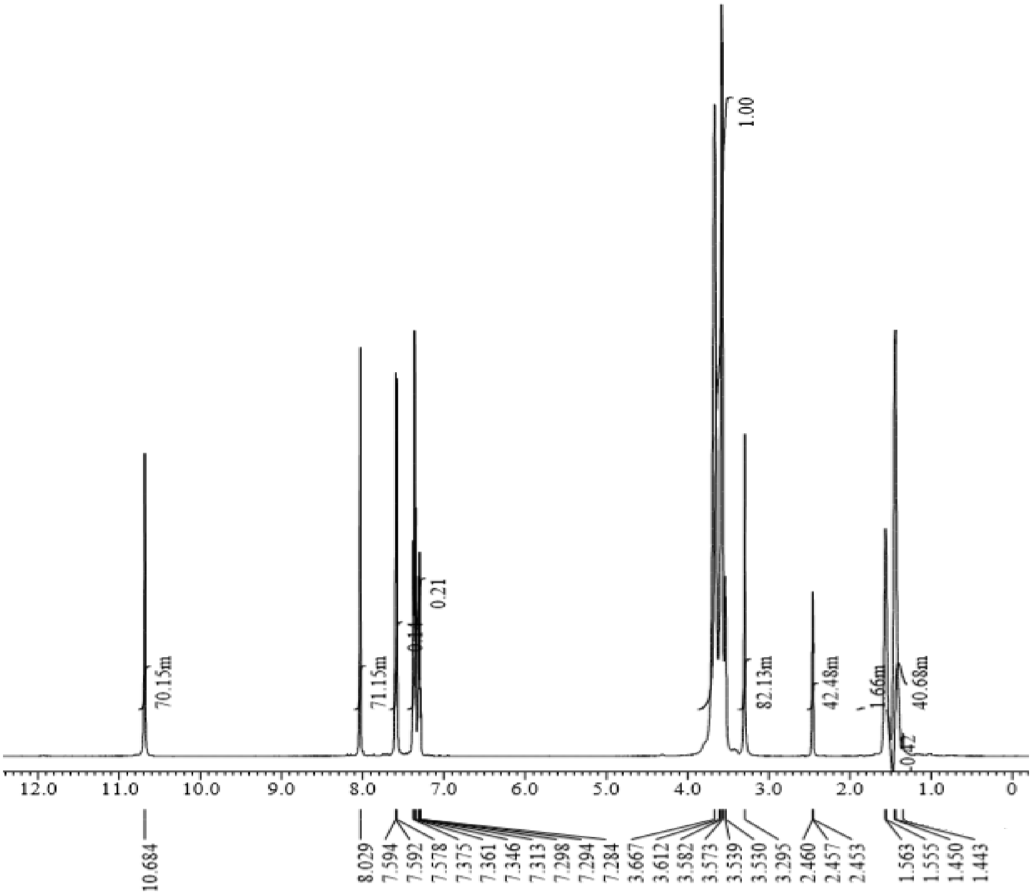

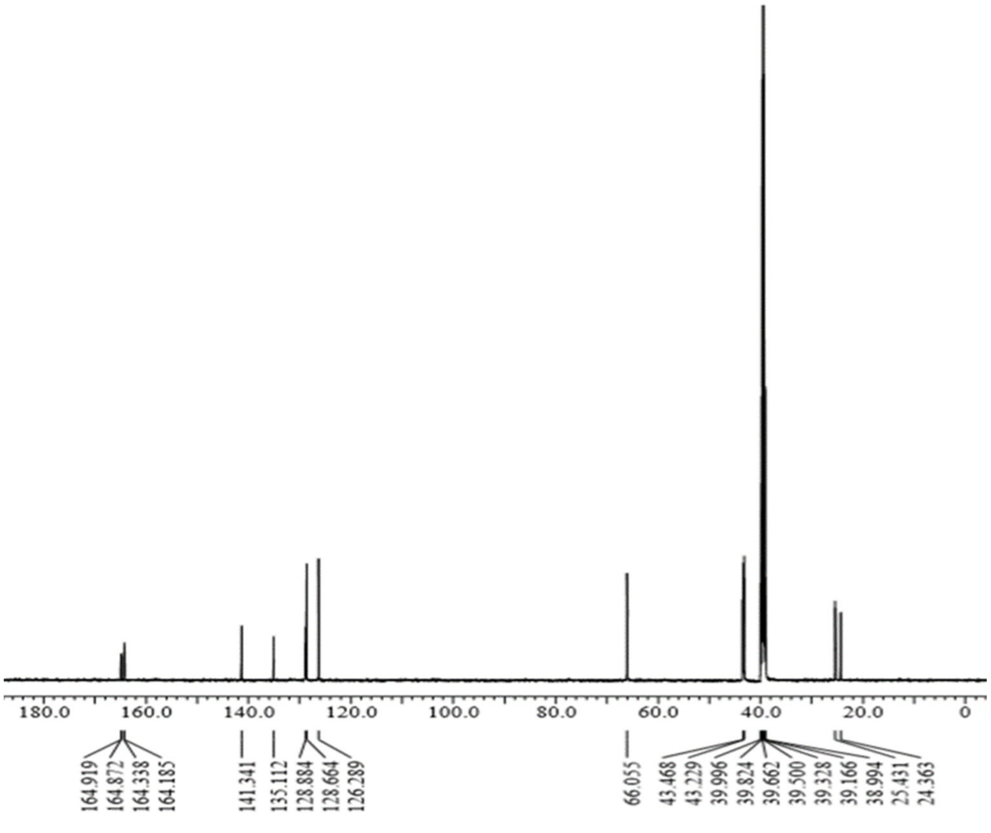

Figure S12. ^{1}H-NMR and ^{13}C-NMR for compound **9a.**

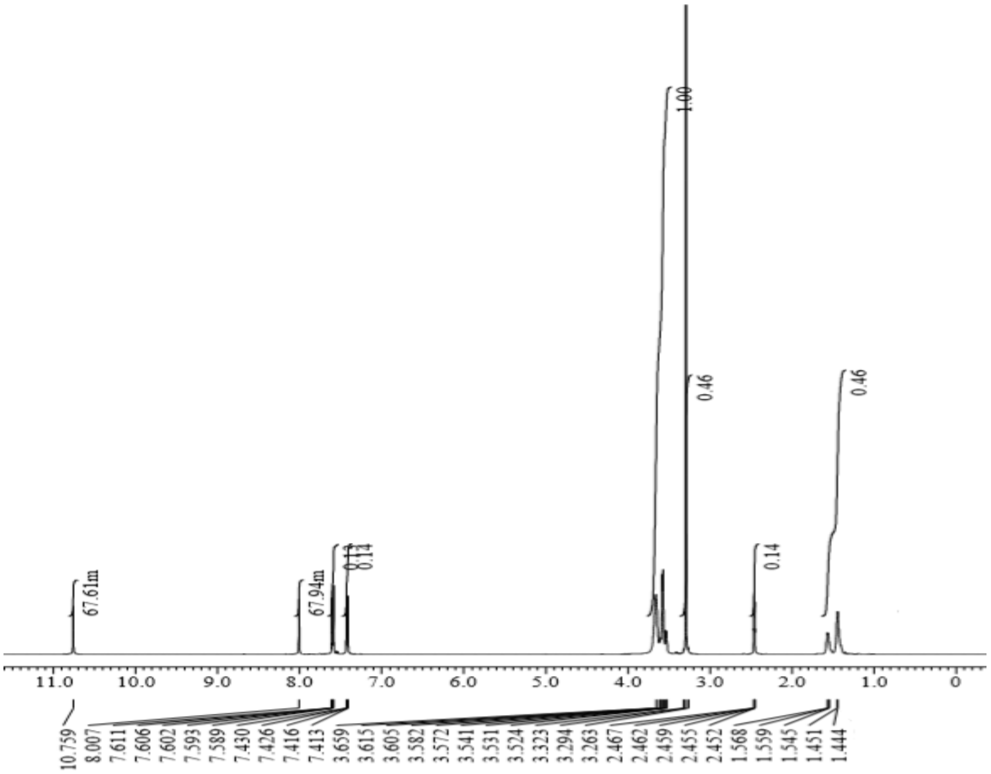

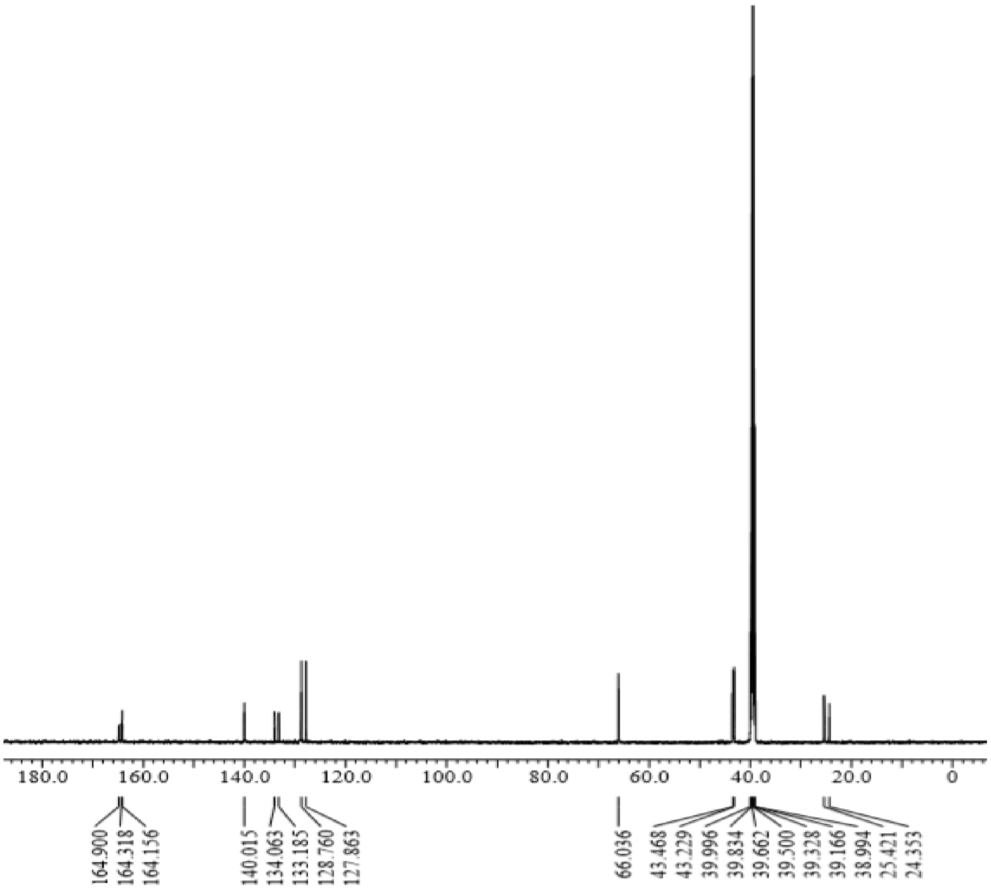

Figure S13. ^{1}H-NMR and ^{13}C-NMR for compound **9b.**

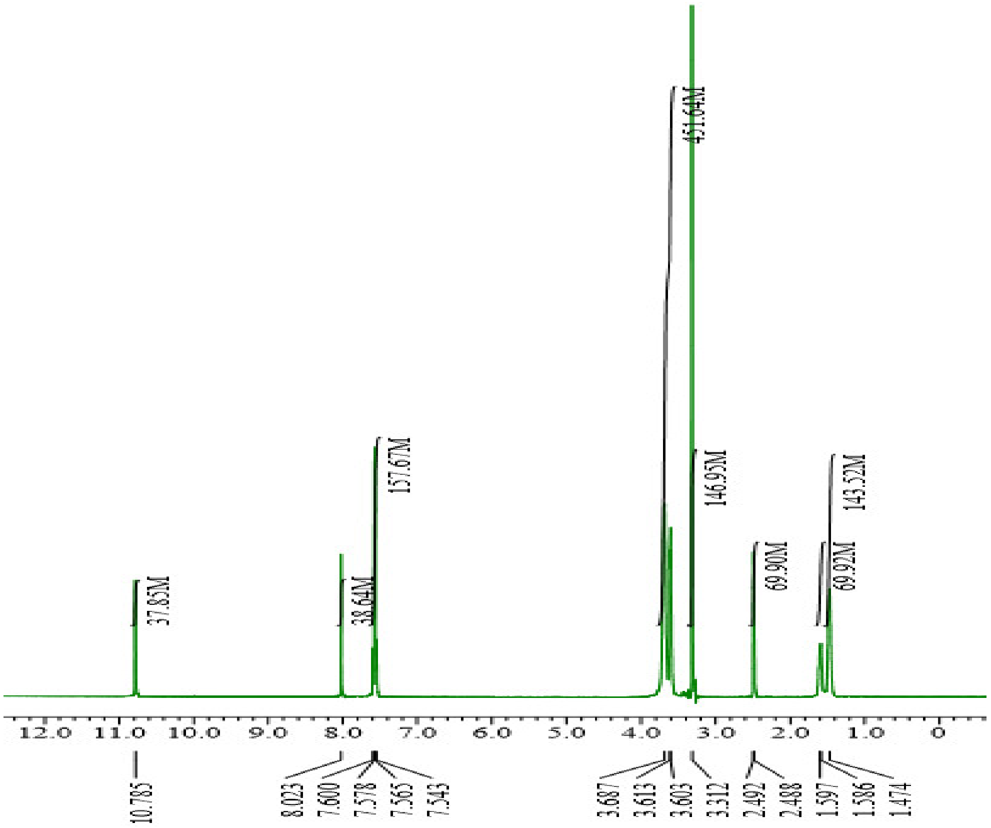

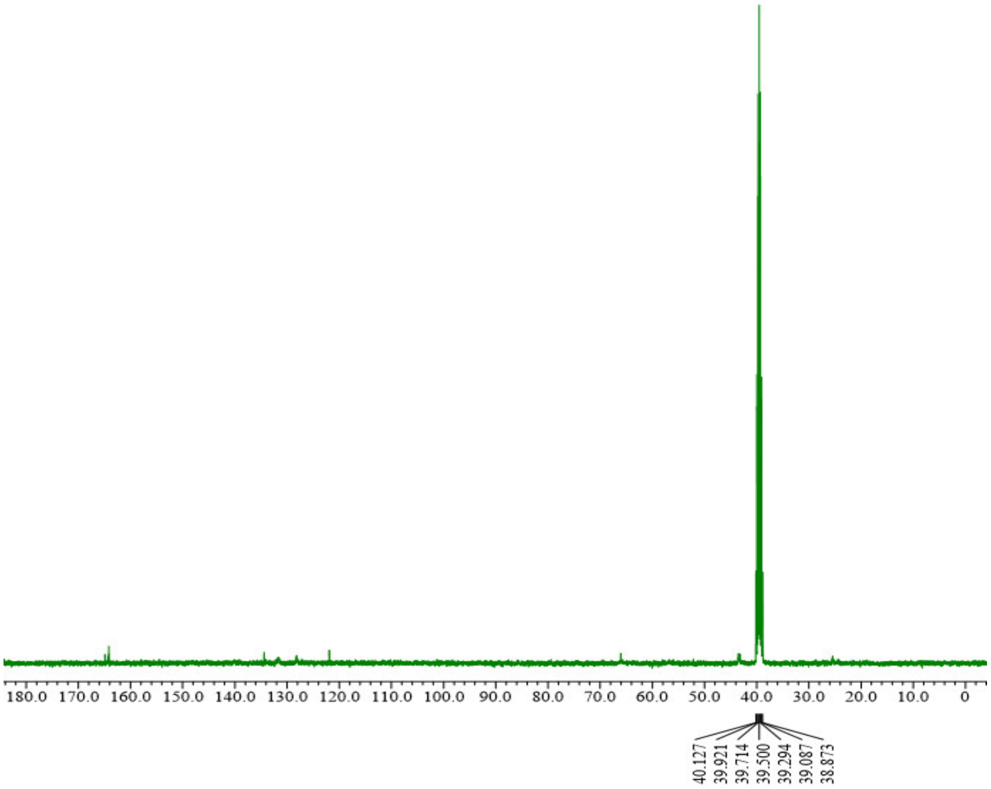

Figure S14. ^{1}H-NMR and ^{13}C-NMR for compound **9c.**

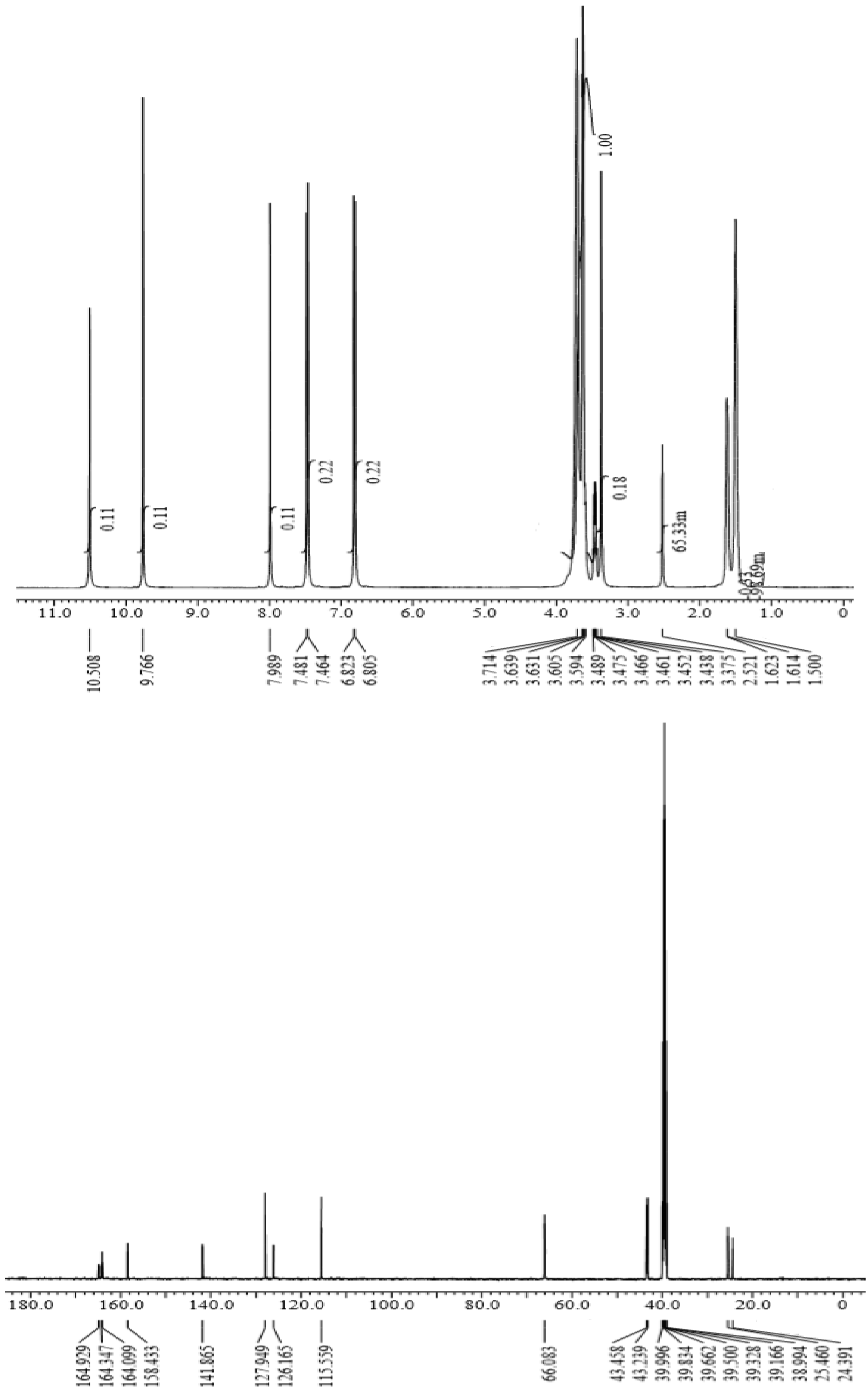

Figure S15. ^{1}H-NMR and ^{13}C-NMR for compound **9d.**

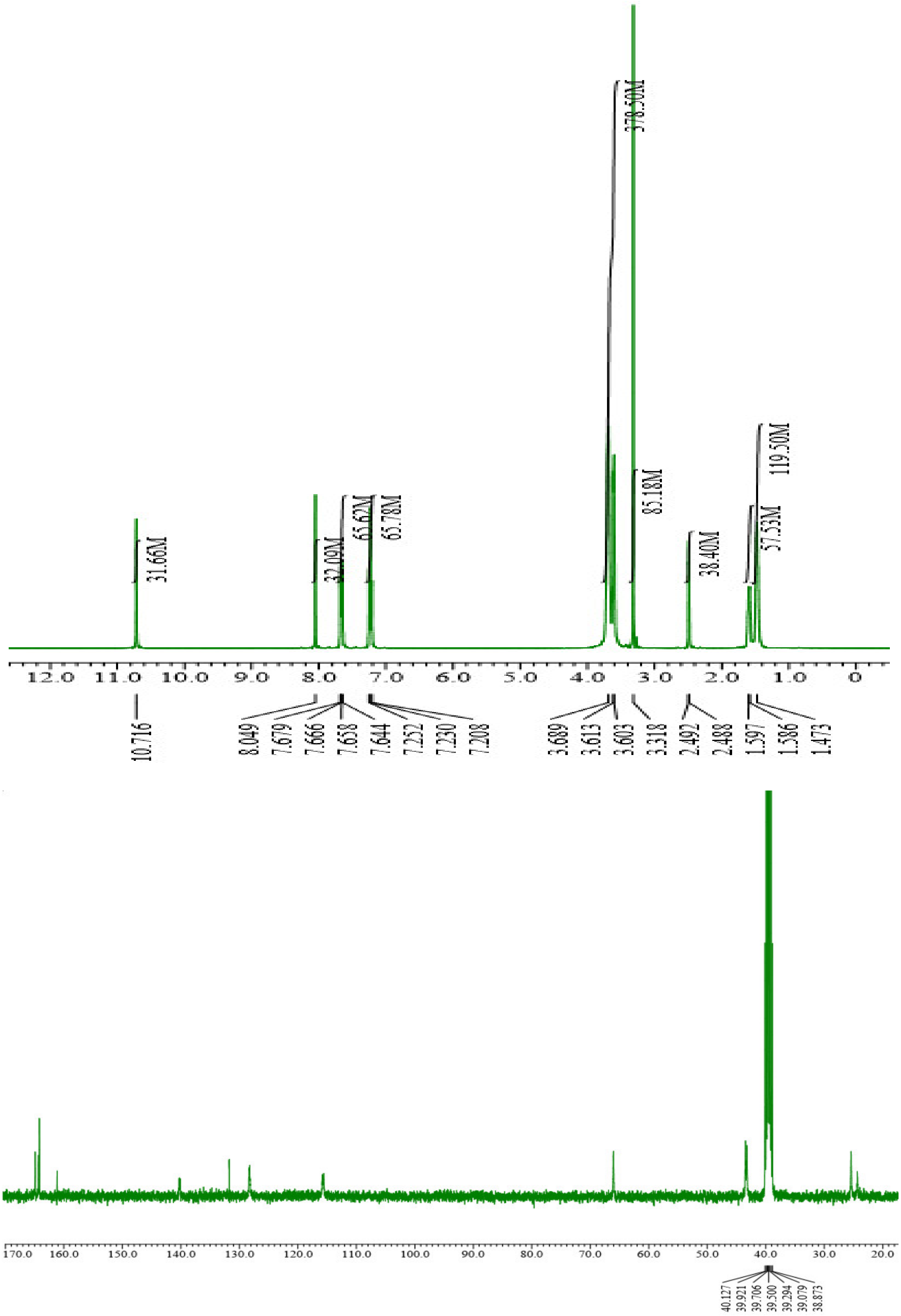

Figure S16. ^{1}H-NMR and ^{13}C-NMR for compound **9e.**

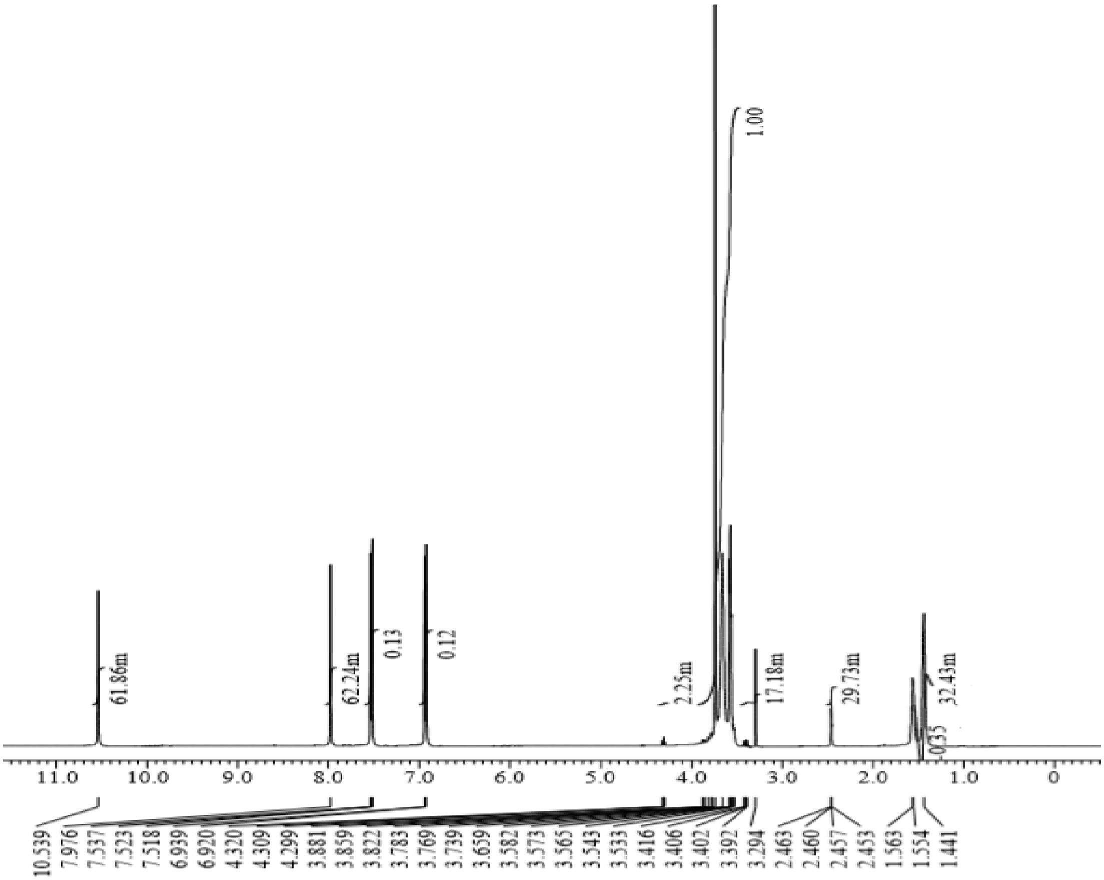

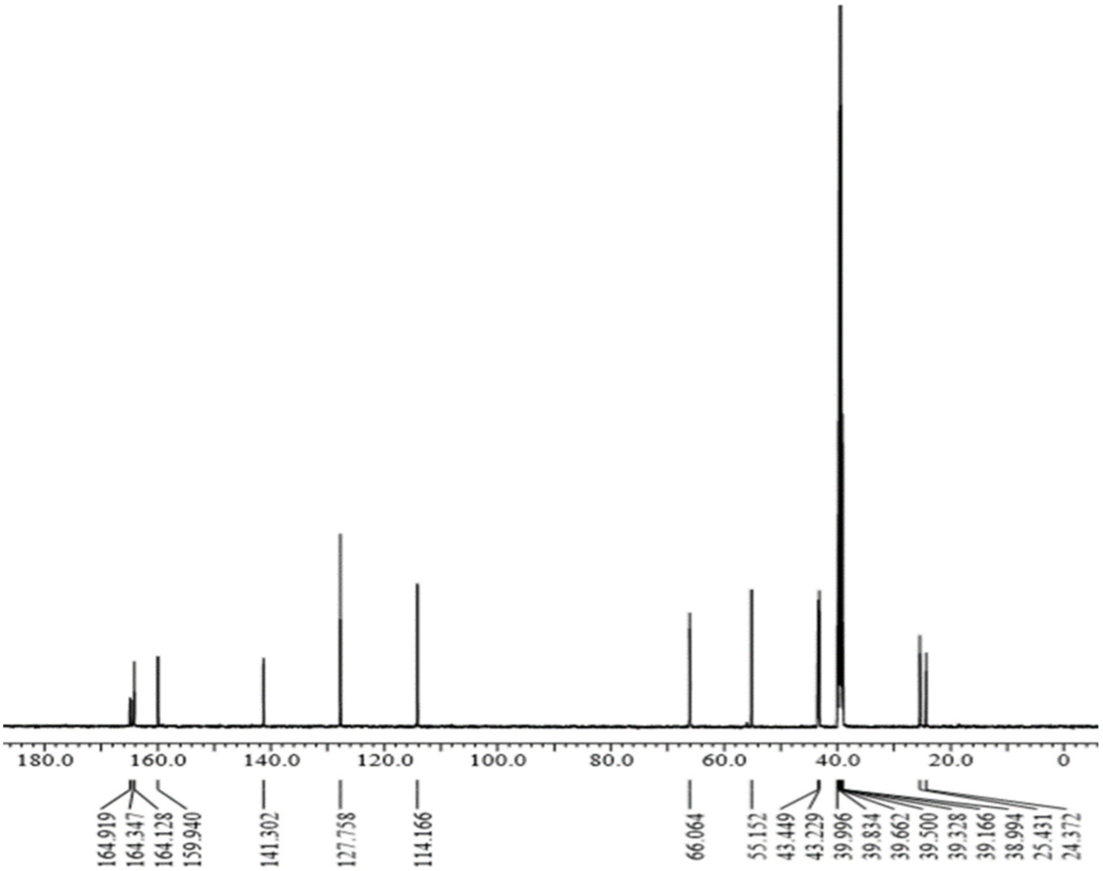

Figure S17. ^{1}H-NMR and ^{13}C-NMR for compound **9f.**

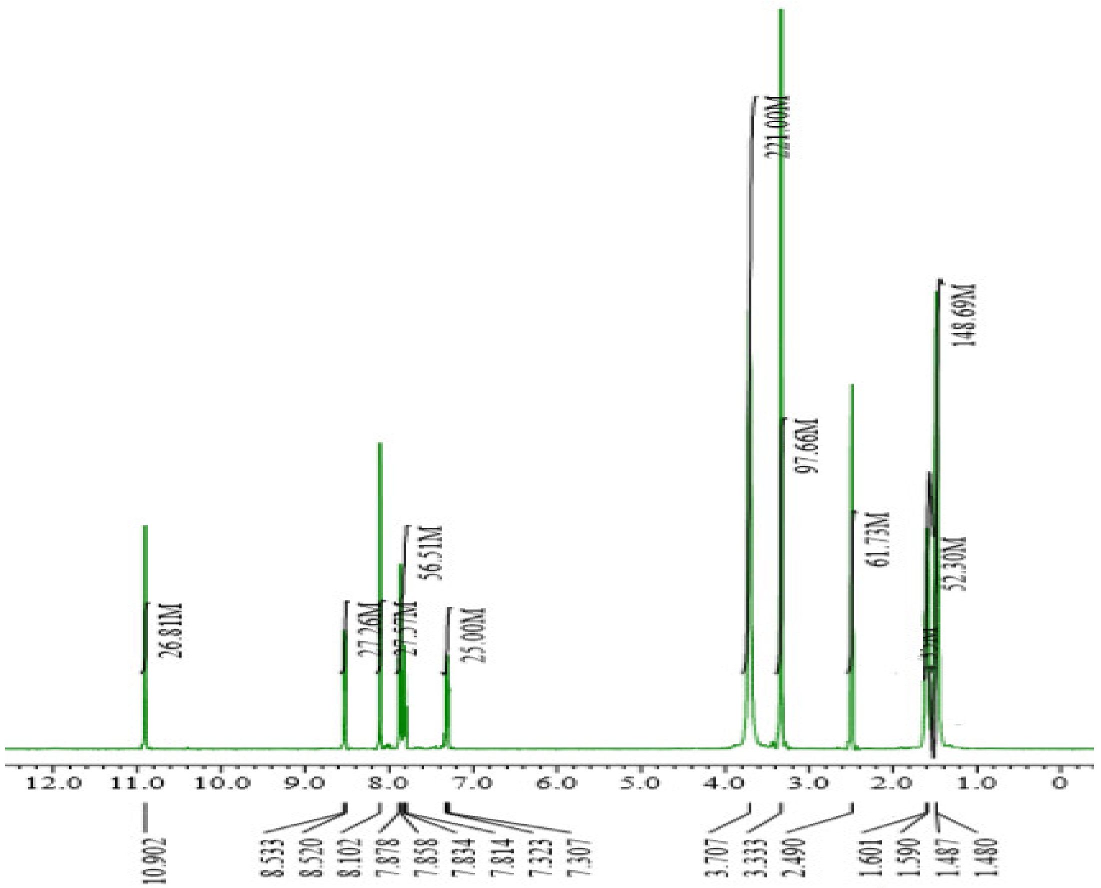

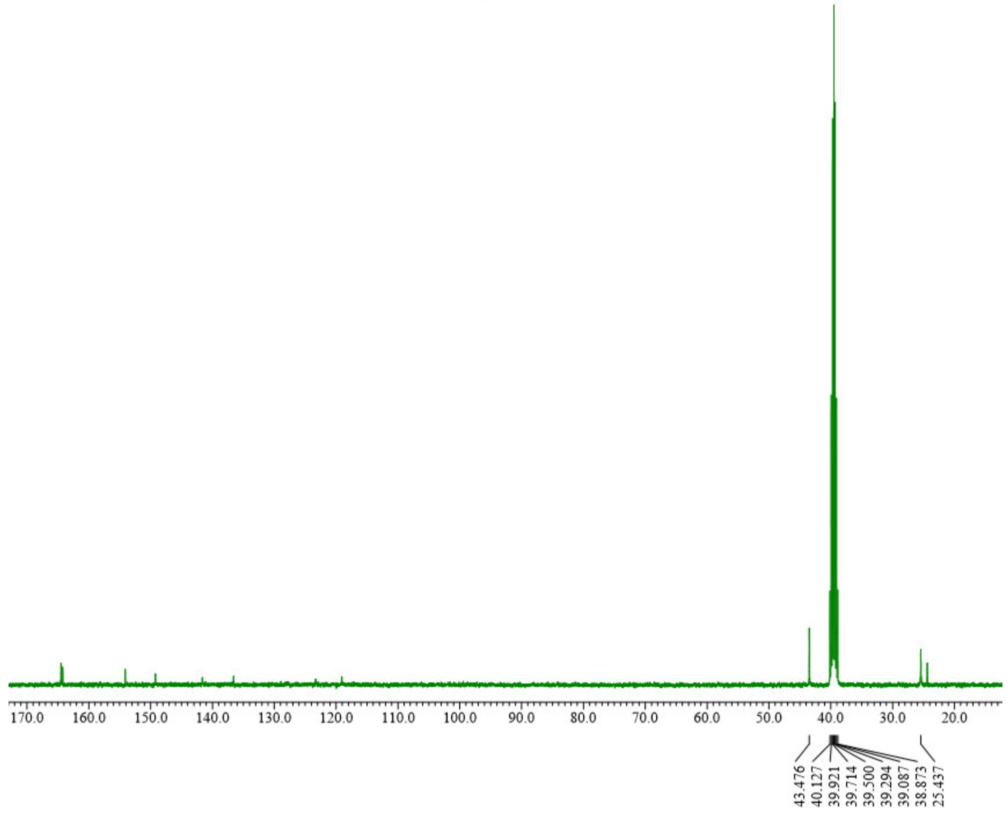

Figure S18. ^{1}H-NMR and ^{13}C-NMR for compound 10.

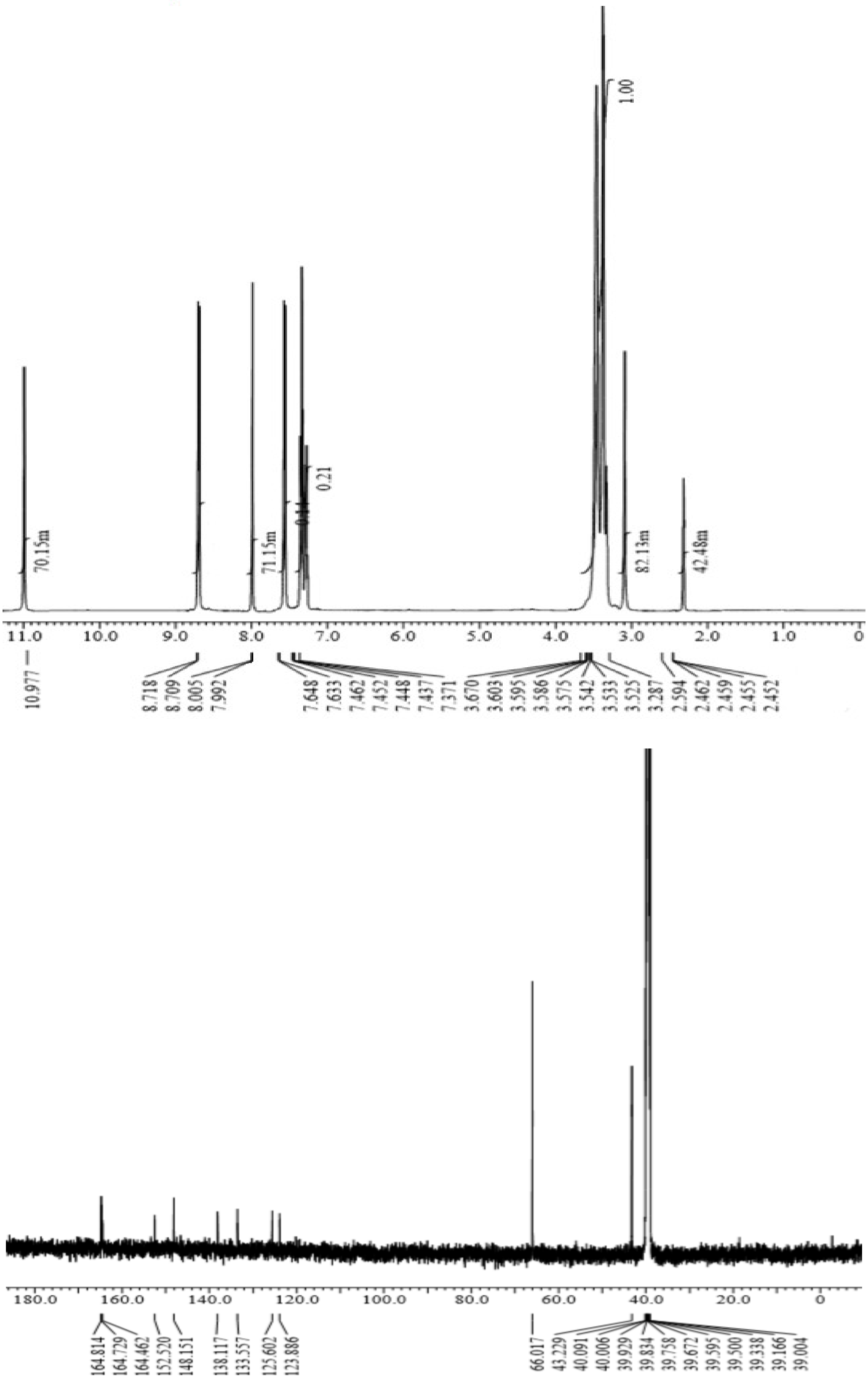

Figure S19. ^{1}H-NMR and ^{13}C-NMR for compound **11.**

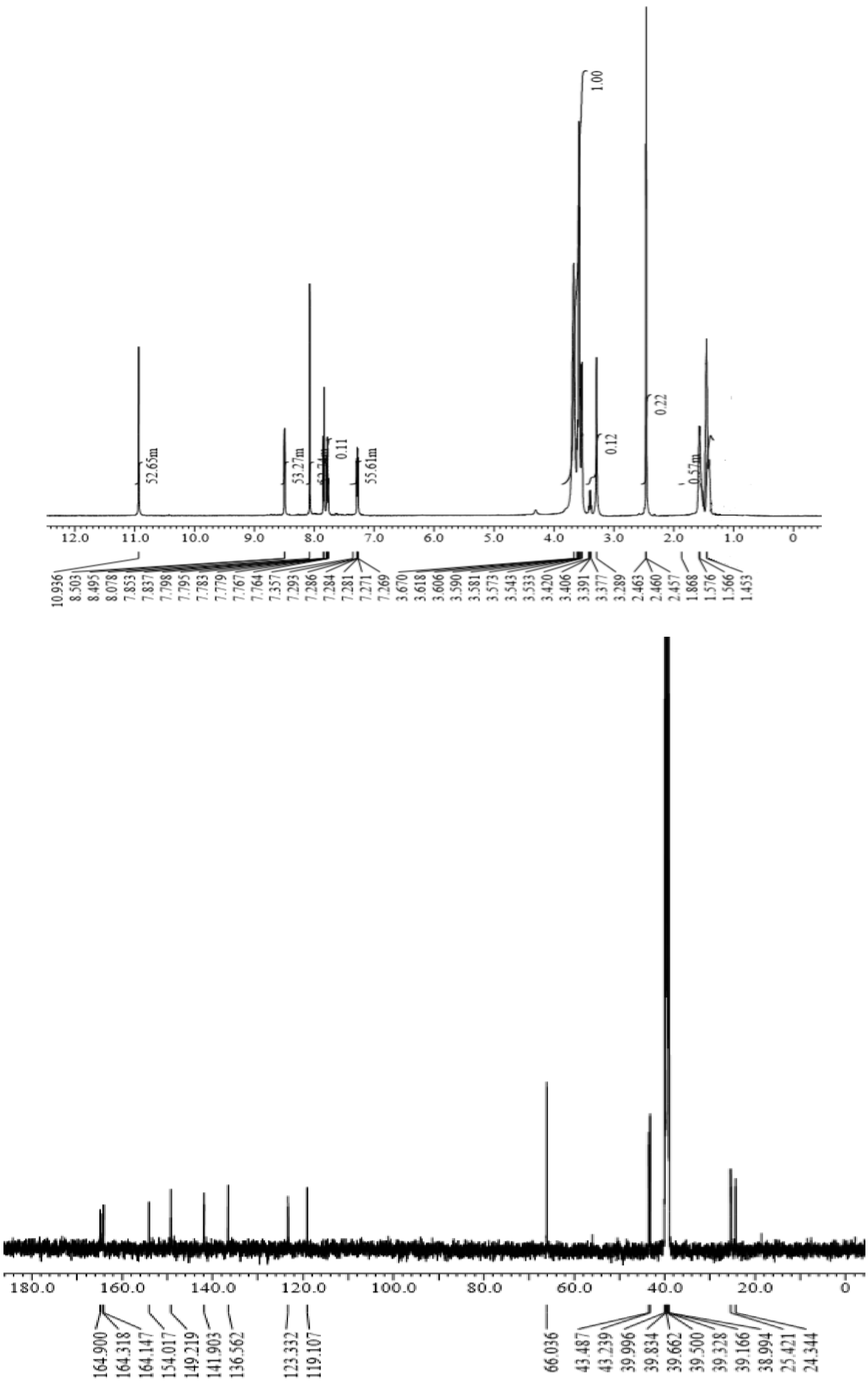

Figure S20. ^{1}H-NMR and ^{13}C-NMR for compound **12.**
